# TCF4 enhances hepatic metastasis of colorectal cancer by regulating tumor-associated macrophage via CCL2/CCR2 signaling

**DOI:** 10.1038/s41419-021-04166-w

**Published:** 2021-09-27

**Authors:** Wei Tu, Jin Gong, Zhenzhen Zhou, Dean Tian, Zhijun Wang

**Affiliations:** 1 Division of Gastroenterology, Tongji Hospital, Tongji Medical College, Huazhong University of Science and Technology, Wuhan, People’s Republic of China; 2 Division of Gastroenterology, Union Hospital, Tongji Medical College, Huazhong University of Science and Technology, Wuhan, People’s Republic of China

**Keywords:** Cancer microenvironment, Cancer models

## Abstract

Colorectal cancer (CRC) liver metastasis is a significant clinical problem for which better therapies are urgently needed. Tumor-associated macrophage, a major cell population in the tumor microenvironment, is a known contributor to primary cancer progression and cancer metastasis. Here, we found TAM recruitment and M2 polarization were increased in the hepatic metastatic lesion compared with the primary site of human CRC tissues. Moreover, Pearson correlation analysis showed that TAM recruitment and polarization were closely correlated with the elevated TCF4 expression in the metastatic site. To investigate the role of TCF4 in CRC liver metastasis, we generated a syngeneic mouse model using MC38 cells splenic injection. Results from in vivo experiments and mouse models revealed that TCF4 deficiency in MC38 cells does not affect their proliferation and invasion; however, it reduces TAM infiltration and M2 polarization in the metastasis site. Further studies indicated that these effects are mediated by the TCF4 regulated CCL2 and CCR2 expression. TCF4 or CCL2 silencing in the tumor cells prevent CRC liver metastasis in the mouse model. Altogether, these findings suggest that the TCF4-CCL2-CCR2 axis plays an essential role in CRC liver metastasis by enhancing TAMs recruitment and M2 polarization.

## Introduction

Colorectal cancer(CRC) is the third diagnosed cancer and one of the leading causes of cancer-related deaths worldwide [1]. Metastasis is the major cause that determines the prognosis of CRC in patients. The liver is the dominant metastatic site for CRC. Up to 30% of patients suffer from liver metastases, and approximately 50%-75% of patients will eventually develop hepatic metastases [2, 3]. To improve CRC patients’ overall survival, we need to further investigate the mechanism of CRC liver metastases and develop novel targets for the clinic therapeutic approaches.

The unique anatomical relationship between the portal vein and mesenteric vein facilitates disseminating gastrointestinal tumors from the primary site to the liver [4]. However, emerging evidence demonstrated that the tumor microenvironment plays an essential role in tumor liver metastasis, including CRC [5–8]. Tumor-associated macrophages (TAMs) represent the majority of immune cells in the tumor microenvironment. Although the macrophage was believed to exert anti-tumor function previously [9, 10], increasing experimental evidence and abundant clinical data suggest that TAMs enhance tumor growth and metastasis [11–14].

It has been well documented that TAMs were involved in all the phases of liver metastasis, defined as pre-metastatic niche formation, microvascular phase, angiogenic, and growth phase [4]. For example, Bruno et al. provided evidence that tumor-derived exosomes educate Kupffer cells to secret cytokines, which in turn promote liver pre-metastatic niche formation, facilitating colonization, angiogenesis, and growth of tumor cells in the liver [15, 16]. TAMs are also considered as an angiogenic switch during tumor progression through the production of VEGF [17, 18]. In addition to these functions, macrophage contributes the immunosuppression through the interaction with immune cells such as cytotoxic T-lymphocyte, NKT cells and B cells. For example, Yang et al. showed that increased PD-L2 expression in TAMs lead to immune evasion and tumor progression through PD-1 signaling [19]. Previous studies have largely enriched our knowledge of TAMs in tumor progression. However, the mechanism of TAMs recruitment and polarization in the tumor microenvironment remains poorly understood.

TCF4, also known as TCF7L2, is an important transcription factor of the TCF/LEF family, which binds to the DNA through the SOX-like HMG domain and enhances target gene expression. There are four members of the family, including TCF1, TCF3, TCF4, and LEF, which are involved in the Wnt pathway [20–24]. In CRC, the activation of Wnt/TCF signaling is considered to be responsible for the onset of cancer development [25, 26]. However, little is known about the role of TCF4 in CRC liver metastasis. In this study, we found TCF4 expression and TAMs recruitment are increased in human CRC hepatic metastases compared with primary sites. However, the relationship between TCF4 expression and TAMs recruitment remains unclear. To this end, we further investigate the role of TCF4 in TMA recruitment and polarization during CRC liver metastasis.

## Materials and methods

### Animals

Eight weeks old male mice with C57BL/6 background were purchased from the Experimental Animal Center of Tongji Medical College, Huazhong University of Science and Technology. (Wuhan, China). All the animal experiments conformed to the guidelines of the Animal Research Ethics Committee of the Huazhong University of Science and Technology. The surgical procedure in this study is performed under a sterile condition in the operation room. All the instruments used were autoclaved before surgery.

### Colorectal cancer patient specimen

All the human samples used in the study were collected from patients with written informed consent. This study was approved by the Institutional Review Board of Huazhong University of Science and Technology.

### Mouse liver macrophage depletion

After anesthetized with 2.5% Isoflurane (300 ml/min), mice were injected with 100 μl (0.05 mg/ml) clodronate liposome (CLO) (clodronateliposomes.org) or PBS liposome via the tail vein. Three days after administration, mice were euthanized for sampling.

### Syngeneic mouse model of colorectal cancer liver metastasis

After anesthetized, a left subcostal incision was made, exposing the spleen by pressing along the cranial and caudal aspects of the incision. Draw up 100 μl of PBS diluted MC38 cells (2 × 10^5^) and injected the cells slowly into the exposed spleen. Located the ligated the spleen blood vessels and removed the spleen 5 min after MC38 cells injection. Close the peritoneum and skin with stitch. 2 weeks after surgery, mice were euthanized for the sampling.

### Orthotopic mouse model of colorectal cancer liver metastasis

A vertical incision was made on the middle abdomen after the mouse was anesthetized by the isoflurane. The abdominal cavity is entered and the cecum with blind-ending pouch was identified and exteriorized. Draw up 50 μl of prepared MC38 cells(2 × 10^5^) with a 27 G syringe and inject the cells into the cecal wall. Carefully inspect the inject site to ensure no leakage after removing the needle from the cecum. Return the cecum to the abdominal cavity and close the incision. After 3 weeks of surgery, the liver was collected for future analysis.

### Primary Kupffer cell isolation and culture

Primary kupffer cells were isolated from mice using two-step perfusion with pronase E and collagenase D, followed by gradient centrifugation using Nycodenz. For the migration assay, KCs were cocultured with MC38 cells as described. For the RNA harvest, 2 × 10^5^ KCs were seeded in the upper chamber with 0.4 μm polycarbonate membrane, 5 × 10^5^ od MC38 cells were seeded in the lower chamber with 10% FBS DMEM. 48 h after coculture, KCs were collected for the RNA extraction.

### Magnetic cell sorting from tumor tissue

Tumor tissue was resected from the liver of the mice model and cut into small pieces of 2–4 mm. Prepare enzyme solution by adding 100 ml of DMEM + 100 mg collagenase D + 2 mg DNase. Transfer the tissue into gentleMACS C tube containing the enzyme solution. Tightly close the C tube and attach it to the gentleMACS dissorciator and continue the digestion program. Resuspend the sample and apply the cell suspension to a 70 μm strainer. Centrifuge the cell suspension at 300 g × 7 min. Resuspend the cell pellet in 90 μl of MACS buffer per 10^7^ total cells. Add 10 μl of antibody MicroBeads per 10^7^ total cells and incubate for 15 min at 4 °C. Wash cells by adding MACS buffer and centrifuge at 300 g for 10 min. Resuspend the cells and proceed to magnetic separation.

### Real-time quantitative PCR

For the Q-PCR, total RNA was isolated from cells or tissue with Trizol(Cat No:15596026, Thermo Fisher) method. The concentration and quality of RNA were tested by the Nanodrop2000. A total of 1 μg of total RNA was reverse transcribed into cDNA using iScript™ Reverse Transcription Supermix(Cat No: 1708841, Bio-rad). Quantitative PCR was conducted using iTaq Universal SYBR Green Supermix(Cat No: 1725120, Bio-rad) on the Bio-Rad CFX96 thermal cycler system. GAPDH was used as an internal reference. The primers used in the study are listed in Table 1.

**Table 1:** Primers.

Mouse primers	Forward 3-5′	Reverse 3-5′
GAPDH	AGGTCGGTGTGAACGGATTTG	GGGGTCGTTGATGGCAACA
TCF4	CGCTGACAGTCAACGCATCTATG	GGAGGATTCCTGCTTGACTGTC
TNF-a	AAGCCTGTAGCCCACGTCGTA	GGCACCACTAGTTGGTTGTCTTTG
iNOS	CGAAACGCTTCACTTCCAA	TGAGCCTATATTGCTGTGGCT
CD86	TCTCCACGGAAACAGCATCT	CTTACGGAAGCACCCATGAT
CD206	CAGGTGTGGGCTCAGGTAGT	TGTGGTGAGCTGAAAGGTGA
VEGF	CGCGAGTCTGTGTTTTTGCA	CAGAGCGGAGAAAGCATTTGT
CD163	GGTGGACACAGAATGGTTCTTC	CCAGGAGCGTTAGTGACAGC
CCL2	ACTGAAGCCAGCTCTCTCTTCCTC	TTCCTTCTTGGGGTCAGCACAGAC
CCR2	ATCCACGGCATACTATCAACATC	CAAGGCTCACCATCATCGTAG
CCR4	TGCACCAAGGAAGGTATCAAGG	GTACACGTCCGTCATGGACTT
F4/80	CTTTGGCTATGGGCTTCCAGTC	GCAAGGAGGACAGAGTTTATCGTG
IL-4	GGTCTCAACCCCCAGCTAGT	GCCGATGATCTCTCTCAAGTGAT
IL-13	CCAGGTCCACACTCCATACC	TGCCAAGATCTGTGTCTCTCC
Human primers	Forward 3-5’	Reverse 3-5’
GAPDH	CATGTTCGTCATGGGGTGAACCA	AGTGATGGCATGGACTGTGGTCAT
TCF4	GGCTATGCAGGAATGTTGGG	GTTCATGTGGATGCAGGCTAC
TNF-a	CAGGCGGTGCCTATGTCTC	CGATCACCCCGAAGTTCAGTAG
iNOS	TTCAGTATCACAACCTCAGCAAG	TGGACCTGCAAGTTAAAATCCC
CD86	CTGCTCATCTATACACGGTTACC	GGAAACGTCGTACAGTTCTGTG
CD206	GCAAAGTGGATTACGTGTCTTG	CTGTTATGTCGCTGGCAAATG
VEGF	AAGGCTGAGCTGGAGGAAG	GGAGCATGATTGAGACTCGC
CD163	TTTGTCAACTTGAGTCCCTTCAC	TCCCGCTACACTTGTTTTCAC
CCL2	CAGCCAGATGCAATCAATGCC	TGGAATCCTGAACCCACTTCT
CCR2	TACGGTGCTCCCTGTCATAAA	TAAGATGAGGACGACCAGCAT
CCR4	CCCACGGATATAGCAGACACC	GTGCAAGGCTTGGGGATACT

### Immunostaining

Paraffin-embedded sections were deparaffinized in xylene and rehydrated in gradient ethanol. Antigen retrieval was performed using a heat-induced method with the citrate-based reagent. After washing with PBS, the slides were blocked with donkey serum for 1 h at room temperature.

For the immunohistochemistry staining, the slides were incubated with rabbit anti-F4/80(Cat No: Ab111101, Abcam), rat anti-CCL2(Cat No: Ab8101, Abcam), and rabbit anti-TCF4(Cat No: 22337-1, Thermo Fisher) primary antibodies at 4 °C overnight HRP-conjugated secondary antibodies for 1 h at room temperature. Next, apply the peroxidase/diaminobenzidine (DAB) substrate solution(Cat No: SK-4100, Vector Lab) to the slides to reveal the color of antibody staining. The positive staining cells were calculated using Image J software(NIH).

For the immunofluorescent staining in mouse samples, the sections were incubated with rabbit anti-F4/80 and rabbit anti-CCR2 primary antibody(Cat No: PA5-23043, Thermo Fisher) 4 °C overnight and incubated with goat anti-rabbit Alexa Fluor 568(Cat No: A11011, Thermo Fisher) and Alexa Fluor 488(Cat No: A32723, Thermo Fisher) for 30 min. The nucleus was stained with DAPI(Cat No: D9542, Sigma).

For the immunofluorescent staining in human samples, the sections were incubated with rabbit anti-CD68 (Cat No: Ab213363, Abcam) and rabbit anti-CCR2 primary antibody(Cat No: PA5-23043, Thermo Fisher) 4 °C overnight and incubated with Alexa Fluor 568 and Alexa Fluor 488 for 30 min. The nucleus was stained with DAPI.

Images of the immunofluorescent staining were visualized by confocal microscopy and applied to the ImageJ software for quantification.

### PCR array

MC38 cells were collected for total RNA extraction using the RNeasy Mini Kit (Cat No: 74104, Qiagen). The quality of the RNA samples was evaluated NanoDrop 2000(Thermo Fisher). Total RNA was reverse-transcribed to cDNA using RT2 HT First Strand Kit(Cat No: 330411, Qiagen). The cDNA samples were diluted 1:10 in nuclease-free water before applying to the PCR Array. To identify the candidate chemokines that are regulated by the TCF4, we performed Mouse chemokine PCR Array(Cat.No. MIMM-116ZD, Qiagen) using miScript SYBR Green PCR Kit(Cat No./: 218076, Qiagen) on the CFX-96 Real-Time System(Bio-Rad). The Quantification cycle values(Cq) were calculated through the Bio-Rad CFX Maestro Software(V4.1). Raw Cq values were uploaded to the Qiagen PCR Array tools (https://dataanalysis.qiagen.com/mirna/arrayanalysis.php) for the subsequent analysis. RNU-6P was determined automatically as the most stable reference miRNA in the array by the tools.

### Promoter activity assay

Luciferase assay was performed in MC38 cells transfected with pCMV-TCF4 plasmid and reporter plasmid(pEZX-FR01) containing wild-type and mutant CCL2 promoter sequence. MC38 cells were seeded in the 96-well plates with a density of 1×104/well and then transfected with indicated plasmids. The culture medium was collected 48 h after transfection and used for the detection of CCL2 promoter activity by the Secrete-Pair™ Dual Luminescence Assay Kit (Cat.No:LF031, Genecopeia).

### Chromatin immunoprecipitation assay

Chip assay was performed in MC38 cells using the Chip kit(Cat.No:17-371, Sigma) according to the manufacturer’s suggested protocol. The DNA fragment was precipitated with TCF4 antibody and anti-mouse IgG protein. The immunoprecipitated DNA fragments were subjected to Real-Time PCR analysis using primers designed from the promoter sequence of CCL2 that contain the TCF4 binding sites.

### Transwell migration assay

A total of 5 × 10^4^ of Kupffer cells were seeded in the upper transwell chamber with 8 μm polyester membrane and cultured with serum-free DMEM. In total 2 × 10^5^ of MC38^CCL2^ and MC38^Control^ cells were seeded in the lower chamber of the 24-well plate and cultured in DMEM with 10% FBS. For the control group, only DMEM with 10% FBS was added to the lower chamber. After 16–24 h of incubation, the membrane of the transwell chamber was fixed and stained with 0.01% crystal violet and observed under the microscope. Migrated cells were calculated using ImageJ.

### Matrigel-transwell invasion assay

A total of 10^4^ of MC38 cells were seeded in the matrigel-transwell chamber with serum-free DMEM. The lower chamber was filled with 10%FBS DMEM. After 24 h of incubation, the lower side of the membrane was fixed and stained with crystal violet. Invaded cells were analyzed by ImageJ.

### Colony formation assay

Transfected MC38 cells were trypsinized and resuspended into single‑cell status by pipetting. 500 MC38 cells were seeded in each well(6-well plate) with 10% FBS DMEM and observed for 2–3 weeks. The cells were then fixed with 4% paraformaldehyde and stained with 0.1% crystal violet for 15 min. Images were taken by the inverted microscope and analyzed using Image J.

### FACS analysis

Isolated cells were trypsinized and blocked for 15 min on ice using mouse FC block(Cat No: 553141, BD) and then incubated with Cell Viability Solution(Cat No: 55815, BD). The resulting cells were incubated with fluorochrome-conjugated antibodies for 1 h. Flow cytometry analysis was performed on the BD Accuri™ C6 Plus Flow Cytometer. Data were analyzed by the Flow Jo software. The antibodies used were listed as follow:

Alexa Fluor 488 anti-mouse CD206 (Cat No: 141709, BioLegend)

Alexa Fluor 594 anti-mouse F4/80 (Cat No: 123140, BioLegend)

### Statistical analyses

All data were presented as mean ± SE. The difference between the two groups was accessed by the unpaired two-tailed Student’s test. One-way ANOVA and Tukey’s post hoc analysis were used to compare the difference in multiple groups. The survival curves in the mice models were analyzed using the Kaplan-Meier method. The correlation between gene expression was analyzed by Pearson’s correlation coefficient test. A p-value of <0.05 was considered statistically significant.

## Results

### TCF4 expression is correlated with TAMs recruitment and M2 phenotype in human CRC tissues

**Fig. 1:**
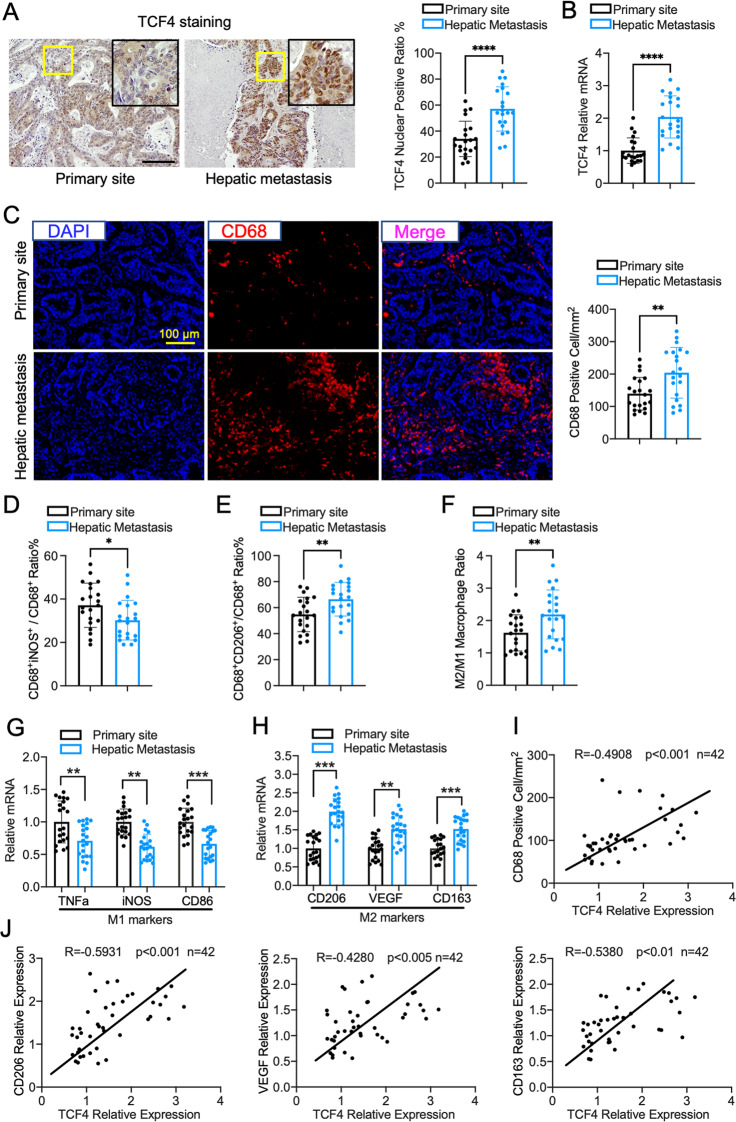
TCF4 expression is elevated in colorectal cancer hepatic metastasis compared with the primary site and is correlated with tumor-associated macrophage recruitment in the tumor area. **A** Representative images and quantification of TCF4 immunohistochemistry staining in human primary colorectal cancer sample and hepatic metastatic sample. *n* = 21 each group; Scale bar, 100 μm; five fields were counted per sample. **B** TCF4 mRNA expression in human samples. **C** Representative immunofluorescence staining and quantification of CD68 expression in human colorectal cancer tissue collected from the primary site and hepatic metastasis. Scale bar, 100 μm; five fields were counted per sample. **D** Quantification of M1 macrophage(CD68^+^iNOS^+^ staining cells)/total TAMs(CD68^+^ staining cells) ratio in human colorectal cancer primary site and hepatic metastases. **E** Quantification of M2 macrophage(CD68^+^CD206^+^ staining cells)/total TAMs(CD68^+^ staining cells) ratio in human colorectal cancer primary site and hepatic metastases. **F** M2/M1 macrophage ratio in human samples. **G** Relative mRNA expression of TNFa, iNOS, CD86 in human colorectal cancer samples. **H** Quantitative PCR results of CD206, VEGF, and CD163 expression in human colorectal cancer samples. **I** Pearson correlation coefficient and p-value between TCF4 mRNA expression and CD68 positive cells density in colorectal cancer samples. (Total *n* = 42; Primary site, *n* = 21; hepatic metastasis, *n* = 21). **J** Pearson correlation coefficient and p-value between TCF4 mRNA expression and CD206, VEGF, CD163 mRNA expression. (Total *n* = 42, Primary site, *n* = 21; hepatic metastasis, *n* = 21). Data are means ± SEM. *P < 0.05, **P < 0.01, ***P < 0.005, ****P < 0.001.

To investigate the role of TCF4 in colorectal cancer liver metastasis, we compared TCF4 expression in human primary colorectal cancer and hepatic metastasis samples. Immunohistochemistry staining showed that TCF4 nuclear staining cells were significantly higher in hepatic metastasis samples than in primary sites (Fig. 1A). In line with the staining results, TCF4 mRNA expression was also increased in the metastatic samples (Fig. 1B). TAMs are an essential component of the tumor microenvironment that enhances tumor growth and invasion, supported by many in vivo and in vitro experiments. The cluster of Differentiation 68(CD68) Is highly expressed in the monocyte lineage, circulating macrophage, and tissue-resident macrophages, including liver Kupffer cells. Therefore, we examined CD68 expression in human colorectal cancer samples. As shown in Fig. 1C, CD68 positive cell density was higher in the metastatic tumor compared with primary sites, which indicated that more TAMs were infiltrated in the hepatic metastasis area.

Despite that infiltrated TAMs numbers in the tumor is essential for the metastatic process and clinical outcome, the phenotype of macrophage is also important [12, 13]. In solid tumors, M1 TAMs exhibit an antitumor function by expressing IL6, IL12, IL23, and TNFa. Conversely, M2 TAMs produce other cytokines including IL4, IL13, IL10, IL-1b, and TGF-b that represent a protumor role. To understand the constitution of different TAMs phenotypes in human primary CRC and hepatic metastases, we stained M1 TAMs with CD68 + iNOS and M2 TAMs with CD86 + CD206 in human samples using immunofluorescence (Fig. S1A, B). Quantification results implied that more M2 TAMs and fewer M1 TAMs were detected in hepatic metastases than primary CRC sites. Further analysis showed that M2 TAMs constituting the main macrophage population in both primary and hepatic metastatic tissue. However, a higher M2/M1 ratio was found in hepatic metastases than primary CRC sites (Fig. 1F).

In addition, we evaluated the expression of M1 and M2 macrophage markers in the tumor samples. Q-PCR results demonstrated that M1 macrophage markers such as TNFa, CD86, and iNOS expressions were decreased in the hepatic metastases (Fig. 1G). In contrast, M2 macrophage markers, including CD206, CD163, and VEGF expressions were higher in metastatic tumors (Fig. 1H). Importantly, Pearson’s correlation analysis indicated that TCF4 expression was correlated with TAMs numbers in the colorectal cancer samples and M2 macrophage markers expression (Fig. 1I, J). Taking together, these findings implied that TCF4 might play a vital role in TAMs recruitment and M2 polarization in the process of colorectal cancer liver metastasis.

### Depletion of macrophage in the liver attenuate CRC liver metastasis

**Fig. 2:**
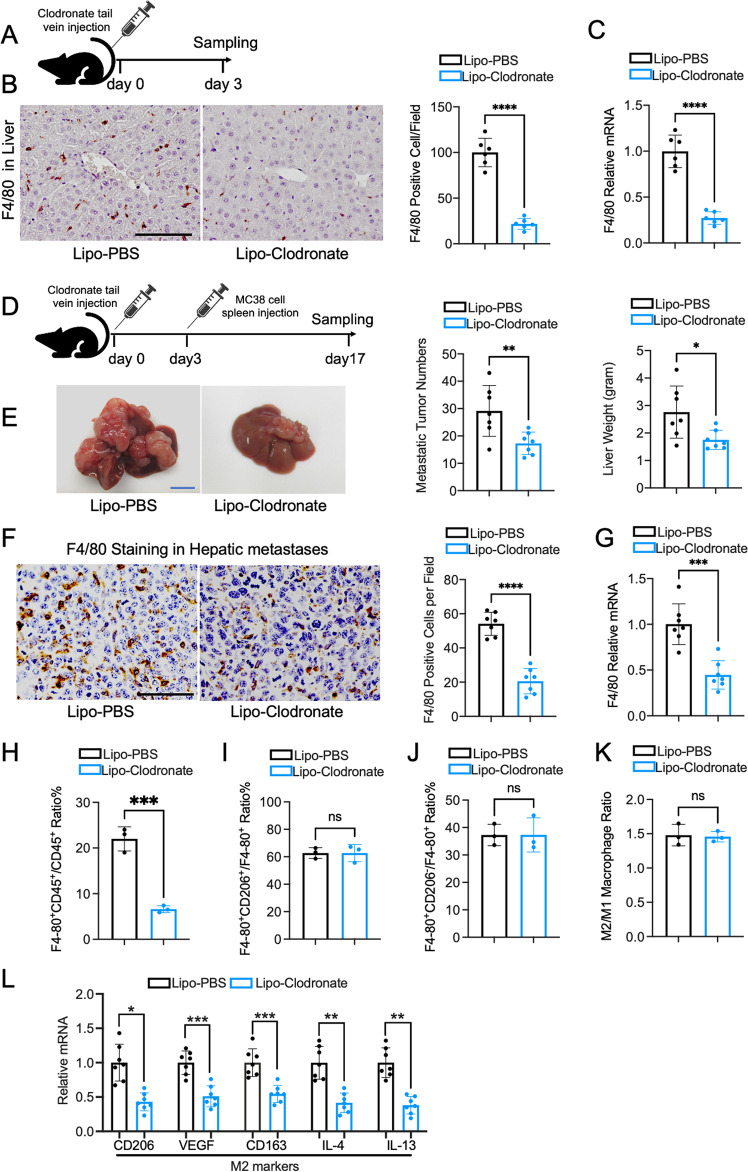
Depletion of macrophage in the liver attenuated colorectal cancer liver metastasis in the mouse model. **A** Schematic protocol of macrophage depletion in the mouse liver using Lipo-Clodronate tail vein injection. **B** F4/80 Immunohistochemistry staining and quantification of Lipo-PBS and Lipo-Clodronate treated mice liver. *n* = 6 each group; Scale bar, 100 μm; five fields were counted per sample. **C** F4/80 mRNA expression in mouse liver. **D** Schematic diagram of colorectal cancer liver metastasis model using liver-macrophage depletion mice. **E** Visible hepatic metastatic tumor nodules and liver weight in mice model. *n* = 7 per group; Scale bar, 10 mm. **F** Representative images and quantification of F4/80 staining in the metastatic tumor area. *n* = 6 each group; Scale bar, 100 μm; five fields were counted per sample. **G** Relative F4/80 mRNA expression in the tumor. **H** Flow cytometry analysis of F4-80^+^CD45^+^/CD45^+^ cell ratio in hepatic metastases of mouse model. **I** Flow cytometry analysis of F4-80^+^CD206^+^/ F4-80^+^ cell ratio in hepatic metastases of mouse model. **J** Flow cytometry analysis of F4-80^+^CD206^-^/ F4-80^+^ cell ratio in hepatic metastases of mouse model. **K** Quantification of F4-80^+^CD206^+^/ F4-80^+^CD206^-^ cell ratio in hepatic metastases of mouse model. **L** M2 macrophage markers(CD206, VEGF, CD163, IL-4, IL-13) mRNA expression level in the tumor. Data are means ± SEM. *P < 0.05, **P < 0.01, ***P < 0.005, ****P < 0.001.

The role of the macrophage in cancer liver metastasis is controversial, depending on factors such as tumor type, stage of metastasis process, tumor size, and interaction with other liver cells. Kupffer cells represent the majority of the liver’s resident macrophages and play an essential role in maintaining liver functions. Several studies suggested that KCs exerted an anti-tumor effect in the early stage when the tumor cells invaded to the liver. For example, depletion of KCs 2 days before tumor injection significantly increased cancer hepatic metastasis. Interestingly, no difference was observed one week after tumor injection [9, 10]. Other studies demonstrated that KC is a crucial component during the formation of tumor pre-metastatic niche, which facilitates the invasion and growth of the primary tumor in the liver [12–14]. To confirm that liver macrophage contributes to colorectal cancer hepatic metastasis, we generated the metastatic model using macrophage-depletion mice [27] (Fig. 1A). Preliminary data showed that three days after tail vein injection, macrophage numbers in the liver are remarkably decreased in mice receiving clodronate-filled liposomes treatment compared with PBS encapsulated liposomes treated group, validated by the F4/80 immunohistochemistry staining (Fig. 2B), Q-PCR (Fig. 2C) and flow cytometry (Fig. S2A and S2B). Moreover, flow cytometry analysis showed that the macrophage population was dramatically reduced in the spleen as well after clodronate-filled liposomes administration (Fig. S2C). Next, to generate the CRC liver metastasis model, mice were subjected to the MC38 cells splenic injection after macrophage depletion (Fig. 2D). Two weeks after spleen injection, the mice were euthanized for the subsequent analysis. As shown in Fig. 2E, hepatic tumor numbers and liver weight was significantly decreased in the macrophage depletion mice compared with control mice. Moreover, we found F4/80 staining and mRNA expression were dramatically dropped in metastatic tumors of the macrophage depletion mice, indicated that TAMs recruitment was attenuated in the tumor area (Fig. 2F, G). In line with the staining and Q-PCR results, flow cytometry analysis indicated that the total macrophage population was reduced in tumor tissue of clodronate treated mice (Fig. 2H). However, clodronate treatment did not impact the F4-80^+^CD206^+^/F4-80^+^ and F4-80^+^iNOS^+^/F4-80^+^ ratio in the tumor area (Fig. 2I, J). Another finding was that M2 TAMs constituting the main macrophage population in hepatic metastases of control and clodronate treated mice (Fig. 2K). This may explain that why tumor growth was inhibited when the total TAMs population was reduced. Besides, the expression of M2 macrophage-associated genes like CD206, CD163, VEGF, IL4, and IL13 was dropped in the tumors of macrophage deficient mice (Fig. 2L). Taken together, these results suggested that depletion of macrophages in the liver prevents the progression of CRC liver metastasis in the mouse model.

### Depletion of TCF4 in MC38 cells impair Kupffer cell migration and M2 polarization in the coculture system

**Fig. 3:**
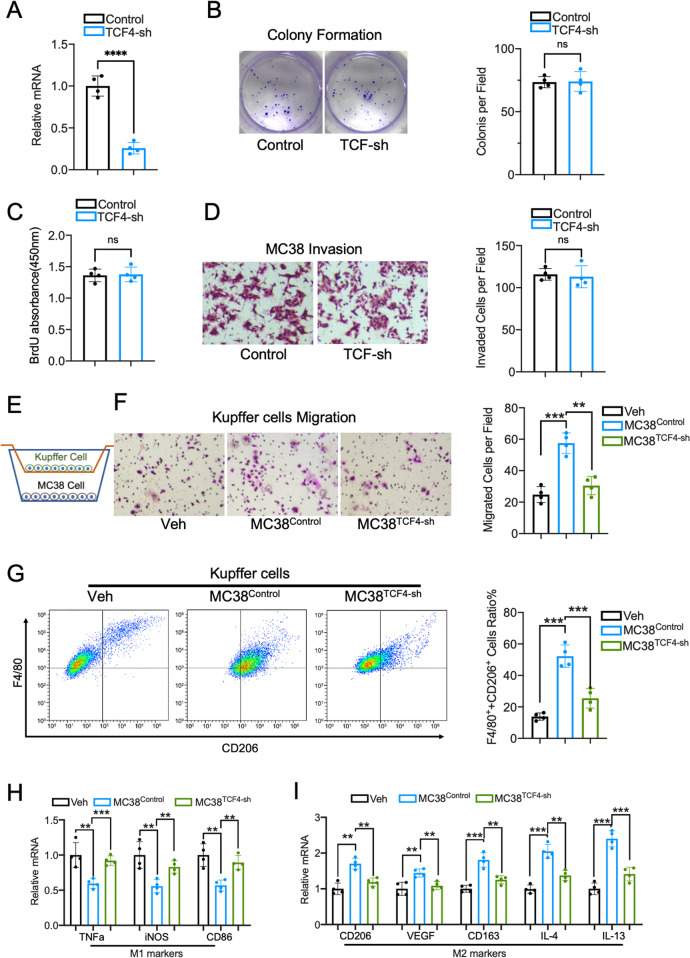
TCF4 is required for the MC38 cells induced Kupffer cell migration and M2 polarization. **A** TCF4 mRNA expression was evaluated in MC38 cells with TCF4-shRNA stable transfection. **B** Representative images and quantification of colony numbers in MC38Control and MC38TCF4-sh cells. *n* = 4 per group. **C** BrdU incorporation assay of MC38 cells. **D** Representative Images and quantification of Matrigel-transwell invasion assay using MC38Control and MC38TCF4-sh cells. *n* = 4 per group; five fields were counted per sample. **E** Schematic diagram of the Kupffer cell and MC38 cell coculture system. **F** Transwell migration assay of Kupffer cells cocultured with indicated MC38 cells or control. *n* = 4 per group; five fields were counted per sample. **G** FACS analysis for F4/80 and CD206 of Kupffer cells co-cultured with indicated cells or control. Quantification of F4/80^+^CD206^+^ cell ratio. *n* = 4 per group. **H** Quantitative PCR results of M1 macrophage markers expression in Kupffer cells. **I** M2 macrophage markers mRNA expression in Kupffer cells cocultured with MC38 cells. Data are means ± SEM. *P < 0.05, **P < 0.01, ***P < 0.005, ****P < 0.001.

Given that TCF4 expression was correlated with TAMs numbers and M2 macrophage-associated gene expression in human CRC samples, we sought to validate the regulating role of TCF4 in macrophage recruitment and polarization. First, we established TCF4 stable knockdown MC38 cells using a lentivirus vector (Fig. 3A). Intriguingly, our data showed that the proliferation and invasion of the MC38 cells were not affected by the TCF4 knockdown as validated by the colony formation assay (Fig. 3B), BrdU incorporation (Fig. 3C), and matrigel transwell invasion assay (Fig. 3D).

Next, we performed a co-culture experiment to illustrate whether TCF4 regulates the communication between MC38 cells and KCs (Fig. 3E). In the liver, resident KCs are the predominant macrophages and are widely studied in the process of tumor hepatic metastasis. Consistent with prior studies, transwell assay and FACS analysis discovered that KCs’ migration (Fig. 3F) and F4/80 + CD206 + ratio (Fig. 3G) were increased when cocultured with normal MC38 cells. Moreover, the expression of M1 macrophage-related genes like TNF-a, iNOS, and CD86 was decreased (Fig. 3H) while the expression of M2 genes including CD206, VEGF, CD163, IL-4, and IL-13 was increased (Fig. 3I). However, these effects on KCs vanished when cocultured with TCF4 deficient MC38 cells, which suggested that TCF4 is essential for the MC38 cells induced TAMs recruitment and M2 polarization. Cancer cells grow in a complicated tumor microenvironment with various stromal cell types, particularly macrophages. Tumor-derived extracellular signals and molecules can influence macrophage’s function and subsequently contribute to tumor progression. To determine the role of M2 TAMs in MC38 cells proliferation and invasion, we next cocultured MC38 cells with or without KCs. As shown in Fig. S3A, B, C, the proliferation and invasion of MC38 cells were enhanced when cocultured with KCs. Moreover,

we found that the growth and invasion of TCF4 deficient MC38 cells were augmented when mixed with wild-type MC38 cells in the co-culture system (Fig. S3D, E, F). Together, these data suggest that TCF4 plays an important role in the interaction between MC38 cells and KCs in vivo.

### TCF4 deficiency in MC38 cells prevent metastatic tumor growth in the liver of mouse model

**Fig. 4:**
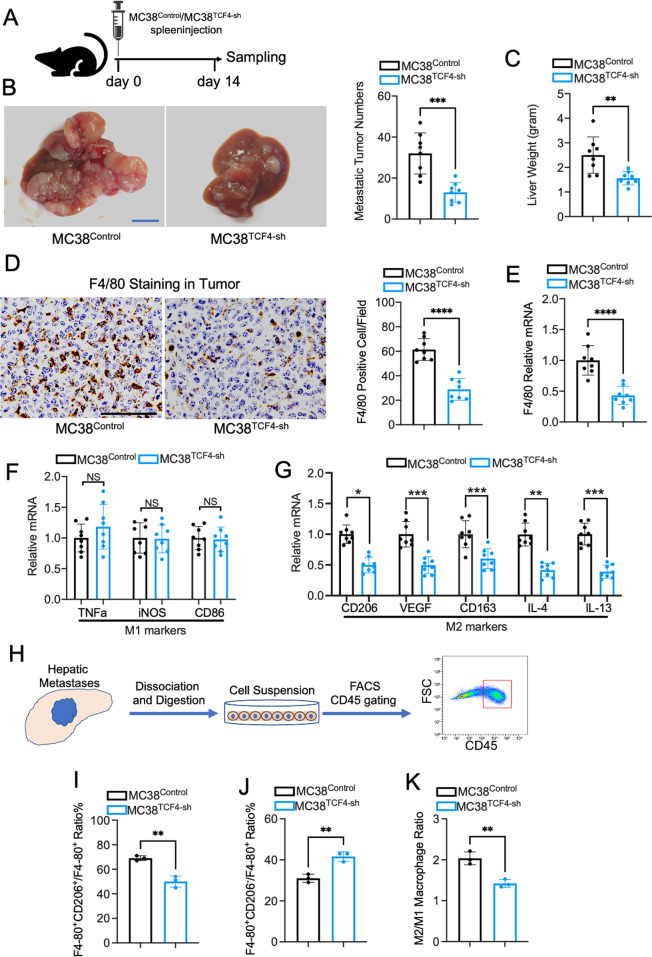
Deficiency of TCF4 expression in MC38 cells significantly reduces TAM recruitment and M2 polarization in the hepatic metastatic tumor of the mouse model. **A** The schematic protocol of the mouse model received MC38 spleen injection. **B** Representative images and quantification of tumor numbers of mouse liver with colorectal cancer metastases. *n* = 8 per group; Scale bar, 10 mm. **C** The liver weight of the mice was evaluated. **D** F4/80 Immunohistochemistry staining and quantification in the metastatic tumor region. *n* = 4 per group; Scale bar, 100 μm; five fields were counted per sample. **E** F4/80 mRNA expression in the tumor was analyzed by Q-PCR. **F** Q-PCR analysis of M1 macrophage marker expression in the tumors. **G** M2 macrophage markers CD206, VEGF, CD163, IL-4, IL-13 mRNA expression in tumors. **H** Illustration of flow cytometry analysis using hepatic metastases tissue of mouse model. **I** M2 macrophage(F4-80^+^CD206^+^)/total TAM(F4-80^+^) ratio in tumors. **J** M1 macrophage (F4-80^+^CD206^-^)/total TAM(F4-80^+^) ratio in tumors. **K** Quantification of M2/M1 ratio in hepatic metastatic tumor of mouse model. Data are means ± SEM. *P < 0.05, **P < 0.01, ***P < 0.005, ****P < 0.001.

To better understand the role of TCF4 in the process of CRC liver metastasis, we generated a mouse model using the TCF4 deficient MC38 cells. As shown in Fig. 4A, the mice were euthanized two weeks after spleen injection using TCF4 deficient and control MC38 cells. Although MC38^TCF4-sh^ cells exert the same proliferation and invasion capacity as MC38^Control^ cells, in vivo data showed that the CRC hepatic metastasis was significantly ameliorated in mice receiving MC38^TCF4-sh^ cells injection compared with MC38^Control^ cells injection (Fig. 4B, C). To characterize how TCF4 impacts the CRC liver metastasis in the mouse model, we evaluated the macrophage infiltration in the hepatic tumors. Immunohistochemistry of F4/80 staining showed that macrophage numbers were reduced in the TCF4 deficient tumors compared with control tumors, which indicated that TAMs recruitment was impaired in the TCF4 deficient condition (Fig. 4D). Consistent with the immunohistochemistry staining results, F4/80 related mRNA expression was dropped remarkably in tumors of mice receiving MC38^TCF4-sh^ cells injection (Fig. 4E). Besides, Q-PCR analysis demonstrated that the M2 macrophage-related gene expression was decreased prominently (Fig. 4F). However, the M1 macrophage-related gene expression showed no difference between the two groups (Fig. 4G). In general, TAMs exhibit an M2 polarization and lead to the progression of tumors; the basal expression of M1 macrophage-related genes in the tumor was significantly lower compared with M2 macrophage. Therefore, despite the fact that the TAMs numbers were decreased, the expression of the M1 macrophage marker showed no difference between the two groups.

Furthermore, flow cytometry analysis showed that TCF4 silencing in MC38 cells significantly reduces M2/Total TAMs ratio(F4-80^+^CD206^+^/F4-80^+^) and increases the M1/Total TAMs ratio(F4-80^+^CD206^-^/F4-80^+^) in hepatic metastases of mice model (Fig. 4H–J). Meanwhile, the M2/M1 ratio in TCF4 deficient tumors was decreased compared with the control tumor (Fig. 4K).

Next, to determine the role of TAMs recruitment and M2 polarization in tumor growth, we have evaluated the Ki67 expression in TCF4 deficient and control tumors by immunohistochemistry and Q-PCR. As shown in Fig. S4A, B, Ki67 expression was significantly downregulated in mice model receiving MC38^TCF4-sh^ cells splenic injection compared with control mice. Consistent with the Ki67 expression, Q-PCR results indicated that CCND1 expression was also decreased in TCF4 deficient tumors (Fig. S4C). In addition, we found invasion related genes including Vimentin, Snail, MMP2, MMP7, and MMP9 were downregulated in TCF4 deficient tumors (Fig. S4D).

To confirm the role of TCF4 in CRC liver metastases, we have performed an orthotopic CRC mice model (Fig. S5A). As shown in Fig. S5B, no macroscopic tumor was observed in both groups 3 weeks after different MC38 cells cecum injection. However, TCF4 depletion in MC38 cells led to a reduction in the incidence of microscopic hepatic metastases (Fig. S5C). Meanwhile, HE staining and quantification of tumor area indicated that TCF4 silencing limits hepatic metastases in the orthotopic model (Fig. S5D). In line with the results from the syngeneic mice model, immunohistochemistry staining and quantification of F4/80 indicated that TAMs recruitment was impaired in hepatic metastases of TCF4 deficient mice model (Fig. S5E).

Taking together, these data indicated that TCF4 expression in the CRC cells is essential for the TAMs recruitment and polarization and subsequent tumor growth in the mice models.

### TCF4 induce TAM recruitment and polarization by regulating the CCL2-CCR2 axis

**Fig. 5:**
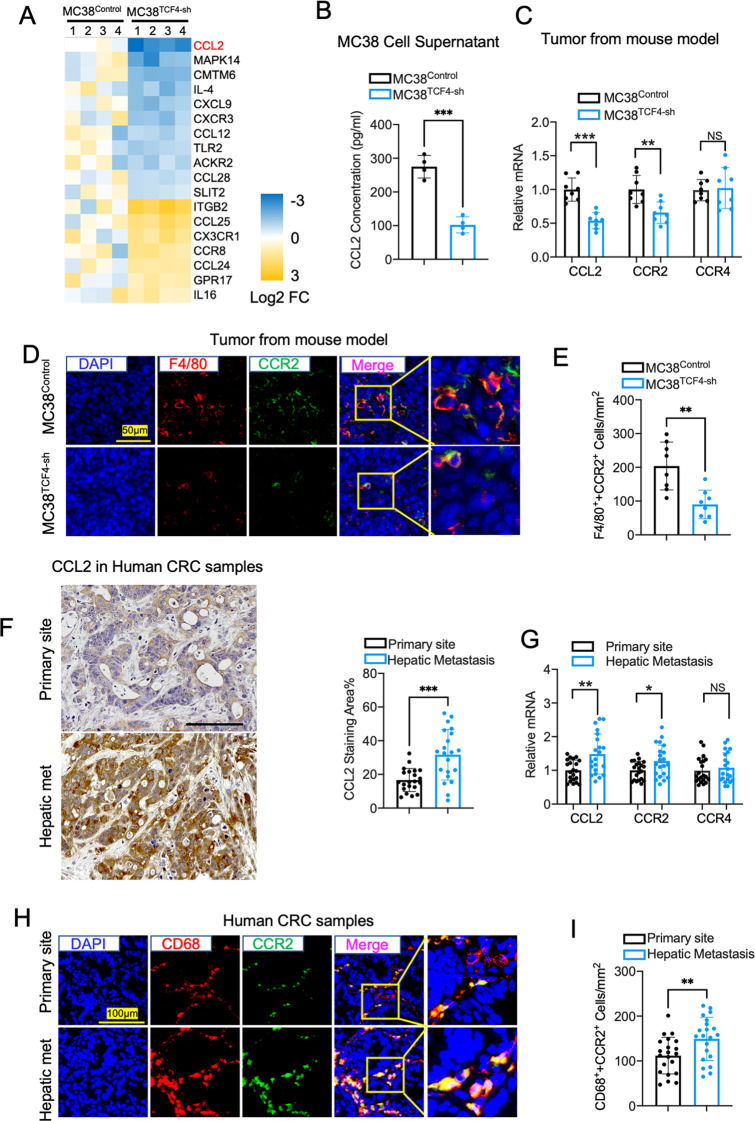
TCF4 enhances TAM recruitment and M2 polarization in the tumor through regulating the CCL2-CCR2 axis. **A** A heatmap of differential expression genes between MC38TCF4-sh cells compared with MC38Control cells using a chemokine PCR array. *n* = 4, per group. **B** CCL2 protein concentrations in the culture medium of MC38Control and MC38TCF4-sh cells were evaluated by ELISA. *n* = 4, per group. **C** CCL2, CCR2 and CCR4 mRNA expression in metastatic hepatic tumors of mice model received MC38Control and MC38TCF4-sh cells spleen injection. *n* = 8 per group. **D** Representative images of Immunofluorescence staining for F4/80(red), CCR2 (green) and DAPI (blue) in mouse hepatic metastases. Scale bar, 50 μm. **E** Quantification of F4/80 and CCR2 double positive cells per mm^2^ tumor area. *n* = 8 per group; five fields were counted per sample. **F** CCL2 Immunohistochemistry staining and quantification in the human primary colorectal cancer and hepatic metastatses. Scale bar, 100 μm. Primary cancer, *n* = 21; hepatic metastases, *n* = 21; five fields were counted per sample. **G** Q-PCR results of CCL2, CCR2, and CCR4 mRNA expression in human primary colorectal cancer and hepatic metastatses. **H** Representative images of Immunofluorescence staining for CD68(red), CCR2(green) and DAPI(blue) in human primary colorectal cancer and hepatic metastatses. Scale bar, 100 μm. **I** Quantification of CD68 + CCR2 + cells per mm2 tumor area. Primary cancer, *n* = 21; hepatic metastases, *n* = 21; five fields were counted per sample. Data are means ± SEM. *P < 0.05, **P < 0.01, ***P < 0.005, ****P < 0.001.

Cancer cell-derived chemokines are responsible for the reprogramming of immune cells in the tumor microenvironment. To gain insight into the mechanism that TCF4 regulates the TAMs recruitment and polarization, we performed a chemokine PCR array using MC38^TCF4-sh^ and MC38^Control^ cells. As demonstrated in Fig. 5A, the expression of several chemokines was altered in MC38^TCF4-sh^ cells compared with MC38^Control^ cells. Notably, we identified CCL2 as the most significantly increased gene. CCL2, as known as monocyte chemoattractant protein 1 (MCP1) and small inducible cytokine A2, is a small cytokine that belongs to the CC chemokine family. CCL2 was a common chemokine that recruits monocytes, memory T cells, and dendritic cells during tumor progression through the CCL/CCR2 and CCL2/CCR4 signaling [28, 29]. First, we evaluated the protein expression of CCL2 in MC38^TCF4-sh^ and MC38^Control^ cells culture medium since CCL2 is a secreted protein. ELISA analysis showed that CCL2 protein was highly expressed in MC38 cells supernatant compared with TCF4 deficient MC38 cells (Fig. 5B). Next, we examined the mRNA expression of CCL2, CCR2, and CCR4 in the hepatic metastases of the mouse model. In comparison with the control tumors, Q-PCR analysis showed that the mRNA expression of CCL2 and CCR2 rather than CCR4 were decreased in the TCF4 deficient tumors (Fig. 5C). Moreover, immunofluorescence staining found that CCR2 expression mostly co-localized with the macrophage marker F4/80 (Fig. 5D). In contrast, the CCR2 + F4/80+ cell numbers were significantly decreased in the TCF4 deficient tumors (Fig. 5E). Consistent with the data from mice samples, the expression of CCL2 and CCR2 was higher in the human hepatic metastases than primary CRC samples validated by immunohistochemistry and Q-PCR (Fig. 5F, G). However, the expression of CCR4 showed no significant difference between two groups. Besides, double immunofluorescence staining demonstrated that more CD68 + CCR2 + cells were observed in the metastatic lesions than the primary site of CRC (Fig. 5H, I). These results suggested that TCF4 might play a role in regulating CCL2/CCR2 signaling, which is positively related to TAM recruitment and enhances the progression of CRC liver metastasis.

### Cancer cell-derived CCL2 contributes to the TAMs recruitment and polarization

**Fig. 6:**
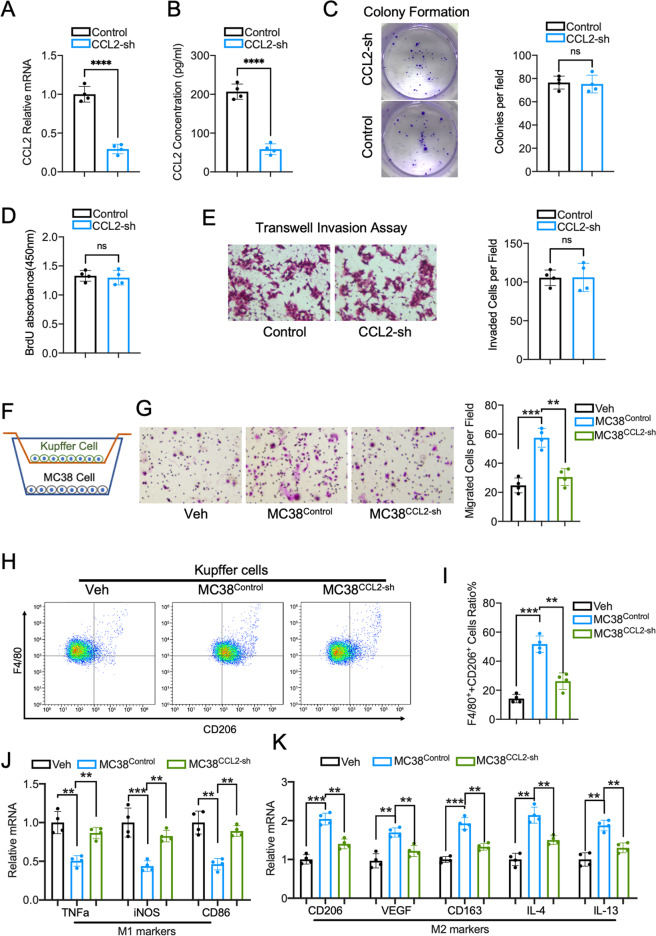
Loss of CCL2 impairs MC38 induced Kupffer cell migration and M2 polarization. **A** TCF4 mRNA expression was evaluated in MC38 cells with TCF4-shRNA stable transfection. **B** CCL2 protein concentrations in the culture medium of MC38Control and MC38CCL2-sh cells were evaluated by ELISA. *n* = 4, per group. **C** Representative images and quantification of colony numbers in MC38Control and MC38TCF4-sh cells. *n* = 4, per group. **D** BrdU incorporation assay of MC38 cells. *n* = 4, per group. **E** Representative Images and quantification of Matrigel-transwell invasion assay for MC38Control and MC38TCF4-sh cells. *n* = 4, per group; five fields were counted per sample. **F** Schematic diagram of the Kupffer cell and MC38 cell coculture system. **G** Transwell migration assay and quantification of the migrated Kupffer cells cocultured with indicated MC38 cells or control. *n* = 4, per group; five fields were counted per sample. **H** FACS analysis for F4/80 and CD206 of Kupffer cells cocultured with indicated cells or control. **I** Quantification of F4/80 + CD206 + cells ratio from three groups. *n* = 4 per group. **J** Q-PCR results of M1 macrophage markers expression in Kupffer cells. **K**. M2 macrophage markers mRNA expression in Kupffer cells cocultured with MC38 cells. Data are means ± SEM. *P < 0.05, **P < 0.01, ***P < 0.005, ****P < 0.001.

Next, we sought to determine whether CRC cells communicate with macrophages by secreting CCL2. To this end, we generated a CCL2 deficient MC38 cell using CCL2-shRNA stable transfection. Q-PCR and ELISA analysis demonstrated that mRNA expression and protein secretion of CCL2 were significantly decreased in MC38^CCL2-sh^ cells compared with control cells (Fig. 6A, B). In addition, we found CCL2 depletion did not affect proliferation and invasion of the MC38 cells using colony formation assay (Fig. 6C), BrdU incorporation (Fig. 6D), and transwell invasion assay(Fig. 6E). However, transwell assay results showed that MC38 induced KCs migration was attenuated by the CCL2 silencing in the MC38 cells (Fig. 6F, G), which implied that CCL2 expression in the CRC cells was essential for KCs recruitment and polarization. Moreover, we found overexpression of CCL2 in the KCs and MC38^TCF4-sh^ coculture system (Fig. S6A) significantly increases migration and M2 polarization of KCs, as demonstrated in Fig. S6B, C. Next, we performed FACS analysis to determine the role of CCL2 on KCs M2 polarization. As showed in Fig. 6H, I, KCs exhibited a remarkable M2 macrophage transition when co-cultured with MC38 cells and this effect was abolished by the CCL2 silencing in the MC38 cells. Furthermore, we evaluated the M1/2 macrophage-related gene expression in the KCs by Q-PCR. Interestingly, M1 macrophage-associated gene expression in KCs was reduced when co-cultured with MC38 cells and restored by CCL2 depletion (Fig. 6J). In contrast, M2 macrophage-related gene expression showed opposite results (Fig. 6K). These in vitro data indicated that MC38 cells secreted CCL2 is essential for macrophage migration and M2 polarization.

### The intervention of CCL2 in MC38 cells attenuates CRC liver metastasis in the mouse model

**Fig. 7:**
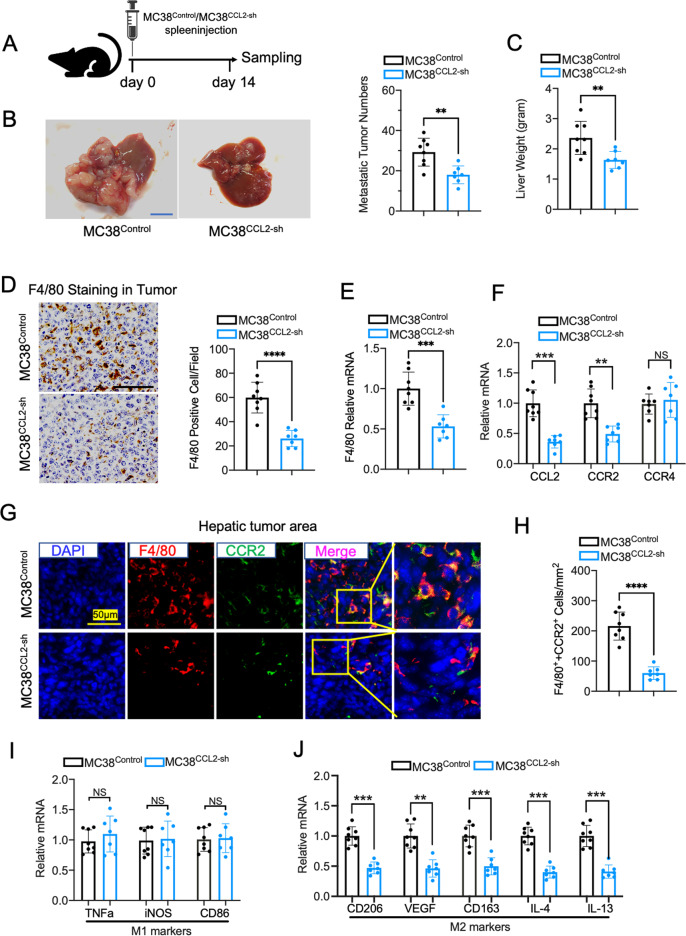
Silencing of CCL2 in MC38 cells inhibits hepatic tumor growth through regulation of TAM recruitment and polarization in mouse model. **A** The schematic protocol of the mouse model received MC38 spleen injection. **B** Representative images and tumor nodule numbers of mouse liver with colorectal cancer metastases. MC38Control group, *n* = 8; MC38CCL2-sh group *n* = 7; Scale bar, 10 mm. **C** The liver weight of the mice was evaluated. **D** F4/80 Immunohistochemistry staining and quantification in the metastatic tumor region. MC38Control group, *n* = 8; MC38CCL2-sh group *n* = 7; Scale bar, 100 μm; five fields were counted per sample. **E** F4/80 mRNA expression in the tumor was analyzed by Q-PCR. **F** Q-PCR analysis of CCL2, CCR2, and CCR4 mRNA expression in the tumors. **G** Representative images of Immunofluorescence staining for F4/80(red), CCR2(green) and DAPI(blue) in mouse hepatic metastases. Scale bar, 50 μm. **H** Quantification of F4/80 and CCR2 double positive cells per mm2 tumor area. MC38Control group, *n* = 8; MC38CCL2-sh group *n* = 7; five fields were counted per sample. **I** mRNA expression of M1 macrophage markers in tumors. **J** M2 macrophage markers CD206, VEGF, CD163, IL-4, IL-13 mRNA expression in tumors. Data are means ± SEM. *P < 0.05, **P < 0.01, ***P < 0.005, ****P < 0.001.

To determine whether MC38 cell-derived CCL2 contributes to the CRC liver metastasis in vivo, we performed a mouse model using MC38^CCL2-sh^ and MC38^Control^ cells spleen injection (Fig. 7A). Two weeks later, the visible tumor numbers and liver weight in mice receiving MC38^CCL2-sh^ cells injection were lower than those of control mice (Fig. 7B, C). Although CCL2 deficiency in MC38 cells did not influence proliferation and invasion in vitro, the lack of CCL2 significantly prevents CRC liver metastasis in vivo.

We next examine the TAMs recruitment in the hepatic metastases using F4/80 immunostaining and Q-PCR. We found a dramatic decrease of TAMs numbers and F4/80 mRNA expression in the CCL2 deficient tumor area compared with control (Fig. 7D, E). To confirm the pivotal role of CCL2-CCR2 signaling in the communication between tumor cells and macrophages, we evaluated CCL2, CCR2, and CCR4 expression in the tumor. Q-PCR results showed that the relative mRNA expression of CCL2 and CCR2 rather than CCR4 was decreased in tumors of mice receiving MC38^CCL2-sh^ cell injection (Fig. 7F). Immunofluorescence staining results showed that CCR2 expression was mostly co-localized with F4/80 positive cells and dropped in the CCL2 deficient tumors due to the reduced TAMs recruitment (Fig. 7G, H). Moreover, we found M2 macrophage-related gene expression was decreased in hepatic metastases of MC38^CCL2-sh^ cells injected mice compared with control mice (Fig. 7J), while M1 macrophage markers expression displayed no difference between the two groups (Fig. 7I). Taking together, these in vivo data suggested that the CCL2-CCR2 signaling was required for the TAMs recruitment and polarization during CRC liver metastasis.

### TCF4 regulates CCL2 expression by enhancing the promoter activity

**Fig. 8:**
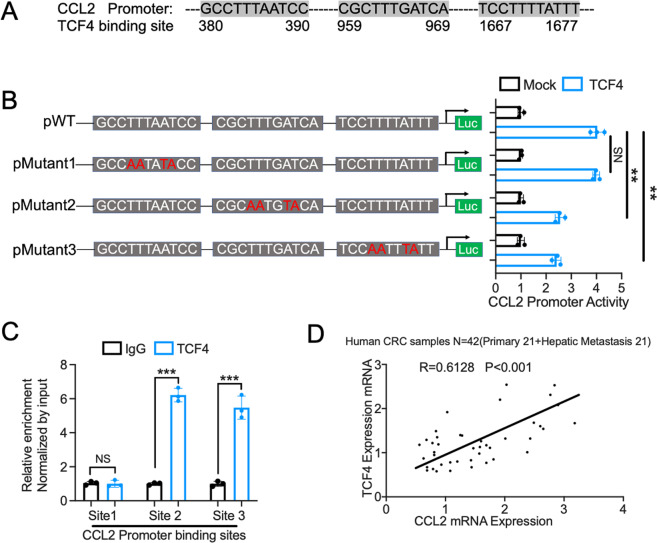
TCF4 regulates CCL2 expression through binding to the promoter region. **A** The promoter sequence of CCL2 contains 3 putative TCF4 transcriptional factor binding sites. **B** Luciferase reporter assays for the activity of wild-type and mutant CCL2 promoters. *n* = 3 per group. **C** Quantitative analysis of ChIP experiments performed on DNA samples precipitated with antibodies against TCF4 and IgG using primers detecting putative TCF4 binding sites on CCL2 promoter. *n* = 3 per group. **D** Pearson correlation coefficient and *p*-value between TCF4 and CCL2 mRNA expression in colorectal cancer samples. (Total *n* = 42; Primary site, *n* = 21; hepatic metastasis, *n* = 21). Data are means ± SEM. *P < 0.05, **P < 0.01, ***P < 0.005, ****P < 0.001.

Given that the TCF4-CCL2-CCR2 axis plays an essential role in TAMs recruitment in the progression of CRC hepatic metastasis, we sought to understand the mechanism of how TCF4 regulates CCL2 expression in MC38 cells. It has been well documented that TCF4 is a transcriptional factor that regulated gene expression by binding to the DNA sequence, especially the gene promoter region. Importantly, we found three putative TCF4 binding sequences in the CCL2 promoter region through the PROMO analysis software (Fig. 8A). To determine whether TCF4 regulates CCL2 expression through binding to the promoter, we conducted site-directed mutation of the putative TCF4 binding sites on the CCL2 promoter. Luciferase assay demonstrated that two of the putative TC4 binding sites are required for the CCL2 promoter activity (Fig. 8). Consistent with the luciferase assay, Chip analysis indicated that TCF4 regulates CCL2 expression through binding to the 959-969 and 1667-1677 motif of the CCL2 promoter region. Furthermore, Pearson’s correlation analysis indicated that TCF4 mRNA expression was correlated with CCL2 mRNA expression in human CRC samples. In summary, these data revealed that TCF4 transcriptionally regulates CCL2 expression by enhancing the promoter activity of CCL2.

## Discussion

CRC liver metastasis starts with disseminating cancer cells from the colon and ends with the clinically detectable lesion in the liver. Once the CRC cells are lodged in the liver, the metastatic tumor secretes cytokines and molecules that induce angiogenesis and proliferation to support further progression [27]. However, the underlying mechanism has yet to be determined. Recent, accumulated studies show that the tumor microenvironment(TME) plays an essential role in cancer metastasis by releasing extracellular signals, promoting tumor angiogenesis, and inducing peripheral immune tolerance [5–7]. Tumor-associated macrophages(TAMs), a critical component of the TME, defined as infiltrated macrophages in tumor tissues, exert multiple functions in tumor metastasis [12–14].

In this study, we found TAMs infiltration was increased in human CRC hepatic metastasis compared with primary tumors. This is consistent with previous clinical findings that the accumulation of macrophages in the TME is associated with a worse prognosis [28–32]. Further analysis indicated that TCF4 expression in the cancer cells is correlated with the TAMs infiltration and M2 polarization in the CRC tumor tissue. Interestingly, silencing of TCF4 expression in MC38 cells did not affect proliferation and invasion of these cells yet influence Kupffer cell migration and polarization using the coculture system. These findings indicated that TCF4 might modulate CRC progression by enhancing the interaction of cancer cells and macrophages rather than promoting proliferation and invasion of the tumor cells directly. However, we still need to identify the downstream molecule that regulates macrophage recruitment and polarization since TCF4 is a transcription factor that can not directly communicate with liver macrophages. Using a chemokine genes PCR array in MC38 cells treated with TCF4-shRNA and control, we identified CCL2 as a potential downstream target. Moreover, our results indicated that TCF4 regulates CCL2 expression in MC38 cells by binding to the promoter region of the CCL2 gene. Immunostaining showed that CRC cells derived CCL2 govern the macrophage infiltration and polarization in the tumor by interacting with CCR2.

CCL2 was considered the leading chemokine expressed by various tumor cells and exerts a pivotal function in immune cell recruitment, especially TAMs. For example, Qian BZ showed that CCL2-CCR2 mediated macrophage infiltration contributes to the distant metastasis of breast cancer, and high CCL2 expression in the tumor predicts poor prognosis in patients [33]. Another study demonstrated that the combination therapy of CCR2 inhibition and Folfirnox improved the outcome of pancreatic cancer in patients [34]. In contrast, Bonapace L showed that discontinuation of anti-CCL2 treatment in mice models significantly accelerates cancer metastasis through regulation of TAMs recruitment [35].

Consistent with previous studies, we found CRC liver metastases were decreased in a mouse model with MC38^CCL2-KD^ cells spleen injection compared with MC38^Control^ cells. Importantly, we found the TCF4-CCL2-CCR2 axis not only contributes to the recruitment of TAMs but also promotes the M2 polarization of TAMs characterized by the increased expression of CD206, CD163, VEGF, IL-4, and IL13. As evidenced by the previous studies, TAMs exhibit diverse functions during the process of tumor metastasis because of the high degree of adaptability in response to the environments [13]. For example, TAMs are essential for the angiogenesis of the tumor area by secreting VEGF and EGF. Moreover, TAMs derived from TGF-beta, PD-L1 and PD-L2 contribute to the depletion of anti-tumor immune cells and lead to the immune escape of tumor cells [19, 36–38]. In line with these findings, our results showed that IL4, IL13, and VEGF expressions were higher in human hepatic metastases than in primary tumors. In vitro and animal experiments found that intervention of TCF4 or CCL2 expression in MC38 cells significantly reduces the production of protumor cytokines in TAMs, indicated that the TCF4-CCL2-CCR2 regulation axis is controlling TAMs polarization.

However, there are limitations to the present study. First, the mouse splenic injection model we used only exhibits the final step of CRC liver metastasis, can not faithfully represent the metastatic pathophysiology. Second, some of the patients included in this study had received chemotherapy, which may influence the immune cells in the tumor samples. Third, although TAMs are one of the most important immune cell populations in the tumor microenvironment, other cell types including neutrophils, NK cells, T-reg, and CD8^+^ cytotoxic T cells also exhibit distinct functions during tumorigenesis [5]. However, in this study, we mostly focused on the role of TAMs in CRC liver metastases. To determine whether TCF4 contributes to the infiltration of other immune cells, we have performed additional flow cytometry analysis using CD45^+^ cells isolated from hepatic metastases of the mice model. As shown in Fig. S7A, B, C, TCF4 depletion does not impact CD11b^+^Ly6G^+^ neutrophils, CD16^+^CD56^+^ NK cells, and CD25^+^FOXP3^+^ T-reg cells population in the tumor microenvironment of syngeneic CRC liver metastases model. In contrast, we found CD3^+^CD8^+^ cytotoxic T cells population was restored in the TCF4 deficient tumors (Fig. S7D). Interestingly, an in vitro co-culture system of CD8^+^ T cells and MC38 cells showed that TCF4 depletion in MC38 cells does not influence CD8^+^ T cells migration, proliferation, and activation (Fig. S7E, F). It is well known that TAMs express an array of effectors that induce immunosuppression during tumorigenesis [13]. We assumed that whether tumoral TCF4 induced TAMs recruitment and M2 polarization contribute to the regulation of CD8^+^ T cell infiltration and activation. To confirm our hypothesis, KCs were first pretreated with wild-type and TCF4 deficient MC38 cells in a co-culture system. The resulting KCs were cocultured with CD8^+^ T cells (Fig. S7G). As expected, wild-type MC38 cells treated KCs significantly inhibits CD8^+^ T cell migration, proliferation, and activation compared with TCF4 deficient MC38 cells treated KCs (Fig. S7H). These results indicated that tumoral TCF4 induced TAMs recruitment and polarization may generate an immunosuppressive, pro-metastatic environment by preventing CD8^+^ T cell infiltration. Fourth, in our study, liver Kupffer cells were used in the in vitro experiments. However, TAMs, at least in mouse models, predominantly originate from immature monocytic cells, which are produced in the bone marrow [39, 40]. In some solid tumors, TAMs also derive from resident macrophage like liver Kupffer cells, which was developed in the yolk sac at the embryonic stage [41–43]. According to the flow cytometry analysis using hepatic metastatic tissue collected from the syngeneic mice model (Fig. S8A), we found CD68^+^CX3CR1^-^ cells constitute approximately 50% of the total CD11b^+^ cell population, while CD64^+^CX3CR1^+^ cells constitute around 30% of total CD11b^+^ cells (Fig. S8B). These results indicated that liver Kupffer cells might be the major source of TAMs population in CRC liver metastases of mice model, which is consistent with some previous studies of solid tumors.

Nonetheless, this study provides evidence that the TCF4-CCL2-CCR2 axis plays an essential role in CRC liver metastasis. Blockade of TCF4-CCL2-CCR2 axis significantly suppressed CRC hepatic metastasis through inhibiting TAMs accumulation and M2 polarization in the tumor microenvironment. Moreover, we demonstrated that tumor cell-derived CCL2 was controlled by TCF4 through binding to the promoter region. Our findings provide a potentially novel mechanism in CRC liver metastasis and may be beneficial for developing agents that target macrophages in the tumor microenvironment.

## Supplementary information

Figure Legend

Figure S1

Figure S2

Figure S3

Figure S4

Figure S5

Figure S6

Figure S7

Figure S8

## Data Availability

The data that support this study are available from the corresponding author upon reasonable request.
