# Supplementary material for: TCF4 enhances hepatic metastasis of colorectal cancer by regulating tumor-associated macrophage via CCL2/CCR2 signaling
Source: Cell Death Dis. 2021 Sep 27;12(10):882. doi: 10.1038/s41419-021-04166-w (PMC8476489; doi:10.1038/s41419-021-04166-w)
Supplement: Supplementary file 1 — Figure Legend [file 41419_2021_4166_MOESM1_ESM.docx]

Figure S1 M1 and M2 macrophage ratio in human primary CRC site and hepatic metastases.

1. Representative images of Immunofluorescence staining for CD68(red), iNOS(green) and DAPI(blue) in human primary colorectal cancer and hepatic metastatses. Scale bar, 100μm.
2. Representative images of Immunofluorescence staining for CD68(red), CD206(green) and DAPI(blue) in human primary colorectal cancer and hepatic metastatses. Scale bar, 100μm.

Figure S2 Clodronate administration reduces macrophage population in mouse spleen, liver, and TAM infiltration in hepatic metastases

1. Illustration of flow cytometry using spleen and liver of clodronate treated mice.
2. Flow cytometry analysis of F4-80^+^CD45^+^ cells in clodronate treated mice spleen.
3. Flow cytometry analysis of F4-80^+^CD45^+^ cells in clodronate treated mice liver.
4. The schematic protocol of FACS analysis using hepatic metastases tissue of clodronate treated and control mice.
5. Flow cytometry analysis of F4-80^+^CD45^+^ cells in hepatic metastases tissue of clodronate treated and control mice.
6. Flow cytometry analysis of F4-80^+^CD206^+^ cells in hepatic metastases tissue of clodronate treated and control mice.

Figure S3 Crosstalk between Kupffer cell and MC38 cells in vitro.

1. MC38 cells were seeded in the co-culture chamber with or without Kupffer cells, and subjected to the following BrdU and transwell invasion assay.
2. BrdU incorporation assay of MC38 cells.
3. Transwell Matrigel invasion assay and quantification of MC38 cells.
4. Protocol of co-culture system using Kupffer cell+MC38^TCF4-sh^ and Kupffer cell+MC38-Mix.
5. BrdU incorporation assay of MC38^TCF4-sh^ cells after co-culture.
6. Transwell Matrigel invasion assay and quantification of MC38^TCF4-sh^ cells after co-culture.

Figure S4 Reduction of TAM infiltration in hepatic metastases limits hepatic metastases proliferation and invasion.

1. Immunohistochemistry staining and quantification for Ki67 in hepatic metastases of mice model.
2. Ki67 relative mRNA expression in mice hepatic metastases tissue.
3. CCND1 relative mRNA expression in mice hepatic metastases tissue.
4. Invasion related genes mRNA expression in in mice hepatic metastases tissue.

Figure S5 Deficiency of TCF4 expression in MC38 cells significantly reduces TAMs recruitment in the hepatic metastatic tumor of the mouse orthotopic CRC model.

1. The schematic protocol of the mouse orthotopic CRC model.
2. Representative images of liver collected from mice model.
3. Incidence of CRC liver metastases 3 weeks after tumor cells cecum injection.
4. Representative images and quantification of liver with metastases.
5. Immunohistochemistry and quantification for F4/80 in hepatic metastases.

Figure S6

1. Recombinant CCl2(and control) was added to the Kupffer cells and MC38^TCF4-sh^ cells coculture system.
2. C. Recombinant CCl2 enhances Kupffer cells migration and M2 polarization in vitro.

Figure S7 Reduction of TAMs recruitment and M2 polarization restores CD8^+^ T cell in hepatic metastases.

A, B, C. Flow cytometry analysis of neutrophils, NKT cell, and T-reg cell in hepatic metastases of mice model.

D. Flow cytometry analysis of CD8^+^ T cell population in hepatic metastases of mice model.

E. CD8^+^ T cell isolated from hepatic metastases were cocultured with MC38^Control^ and MC38^TCF4-sh^ cells.

F. TCF4 depletion in MC38 cells does not influence proliferation, migration and activation of CD8^+^ T cell in coculture system.

G. Kupffer cells were cocultured with MC38^Control^ and MC38^TCF4-sh^ cells. 24 hours after coculture, Kupffer cells were cocultured with CD8^+^ T cells.

H. CD8^+^ T cells were subjected to the migration, BrdU, and RT-PCR assay after cocultured with different Kupffer cells.

Figure S8 Both Kupffer cells and BMDM contribute to the infiltrated TAMs population in hepatic metastases of mice model.

1. Schematic protocol of flow cytometry analysis.
2. Kupffer cells are the major source of TAMs in hepatic metastases in mice model.
