# Supplementary figures and images for: TCF4 enhances hepatic metastasis of colorectal cancer by regulating tumor-associated macrophage via CCL2/CCR2 signaling
Source: Cell Death Dis. 2021 Sep 27;12(10):882. doi: 10.1038/s41419-021-04166-w (PMC8476489; doi:10.1038/s41419-021-04166-w)

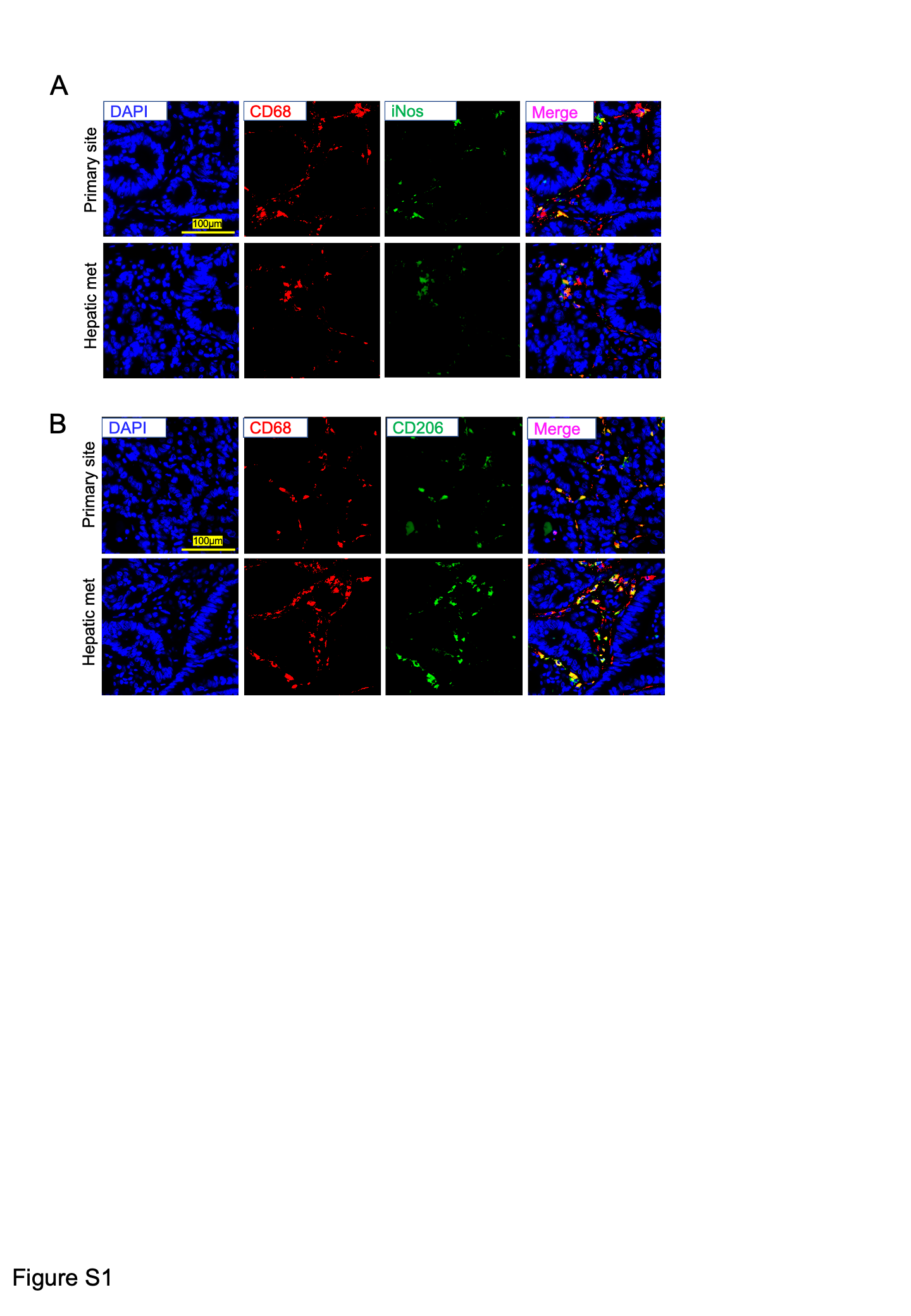

Supplement: Supplementary file 2 — Figure S1 [file 41419_2021_4166_MOESM2_ESM.tif]

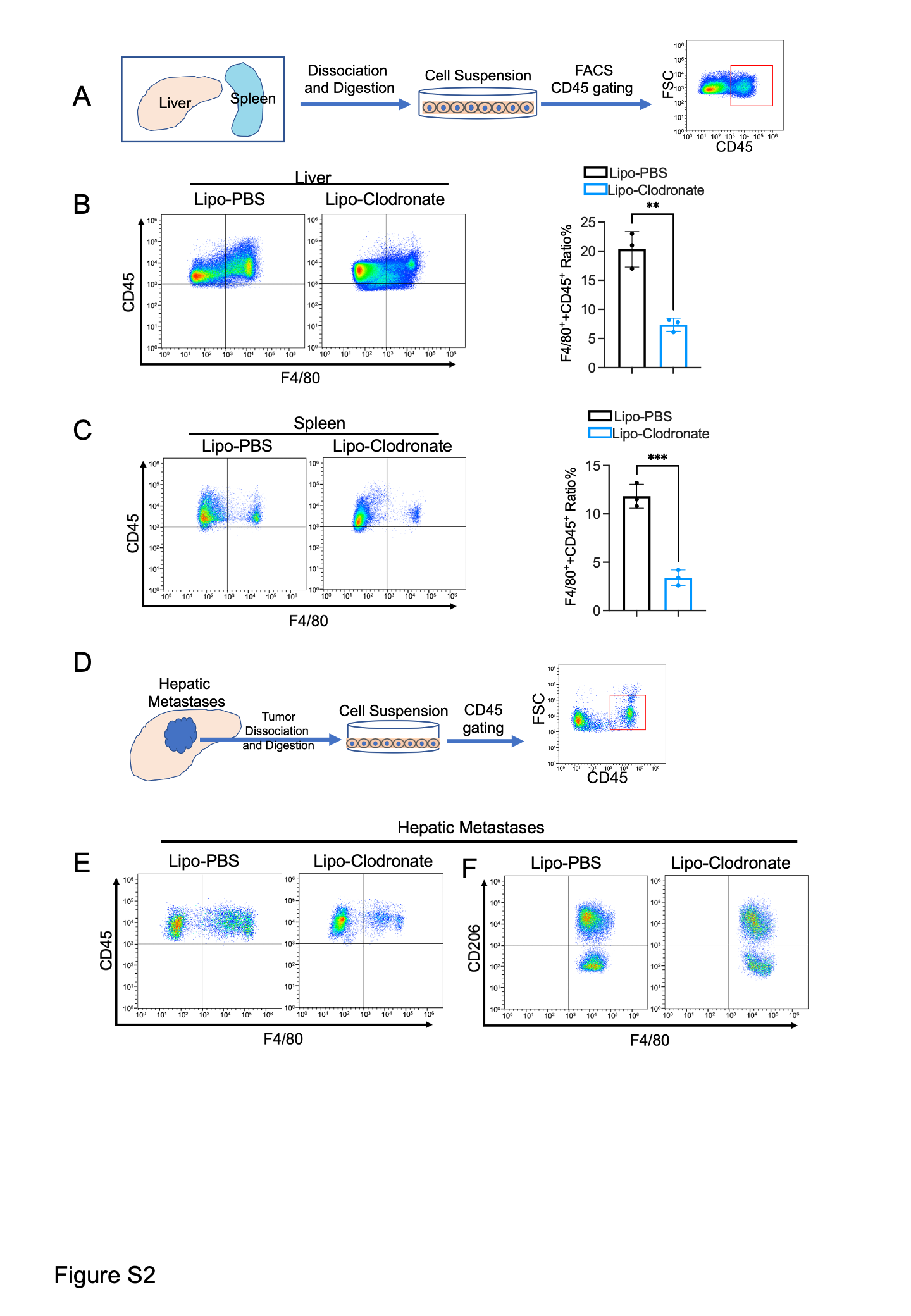

Supplement: Supplementary file 3 — Figure S2 [file 41419_2021_4166_MOESM3_ESM.tif]

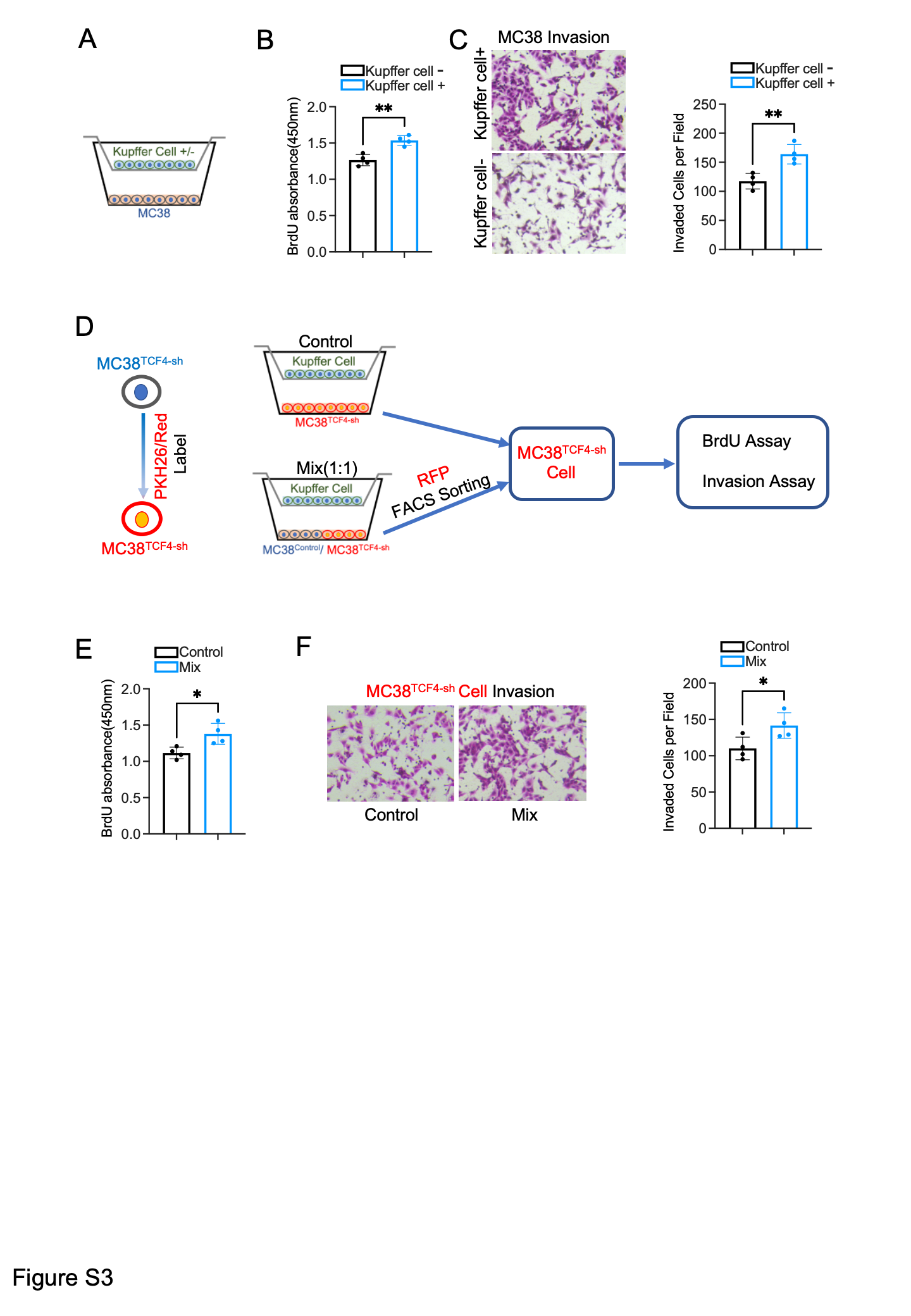

Supplement: Supplementary file 4 — Figure S3 [file 41419_2021_4166_MOESM4_ESM.tif]

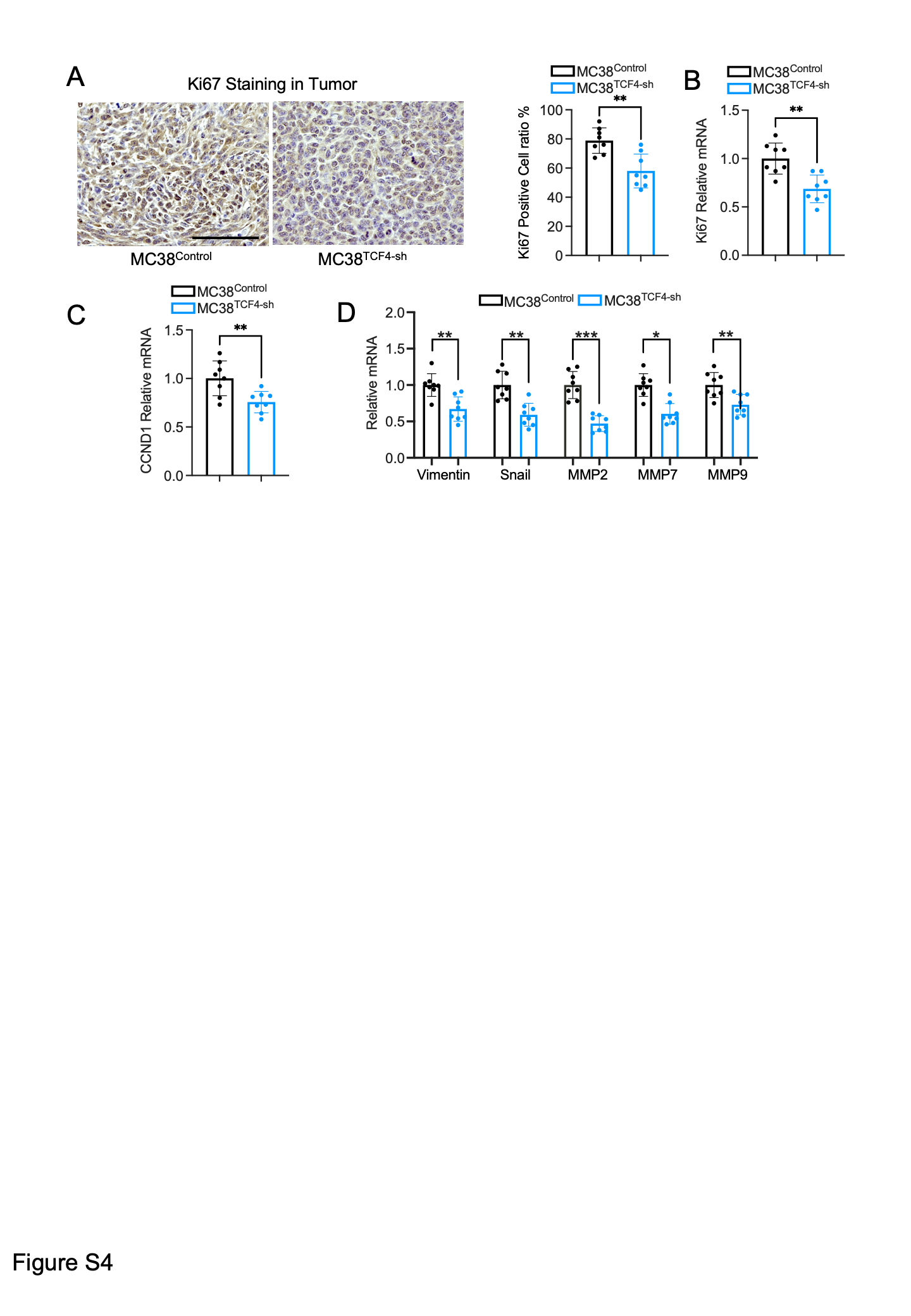

Supplement: Supplementary file 5 — Figure S4 [file 41419_2021_4166_MOESM5_ESM.tif]

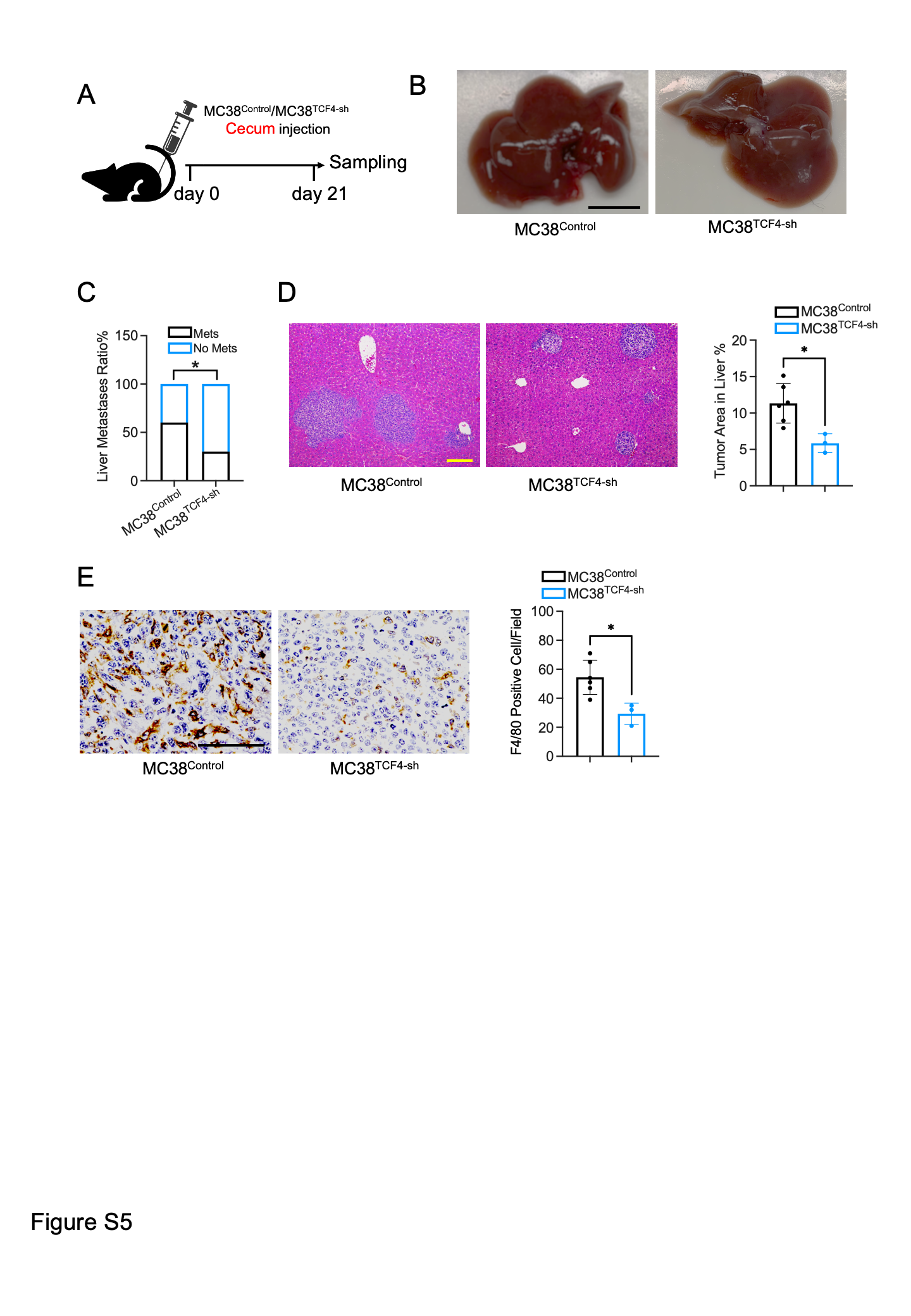

Supplement: Supplementary file 6 — Figure S5 [file 41419_2021_4166_MOESM6_ESM.tif]

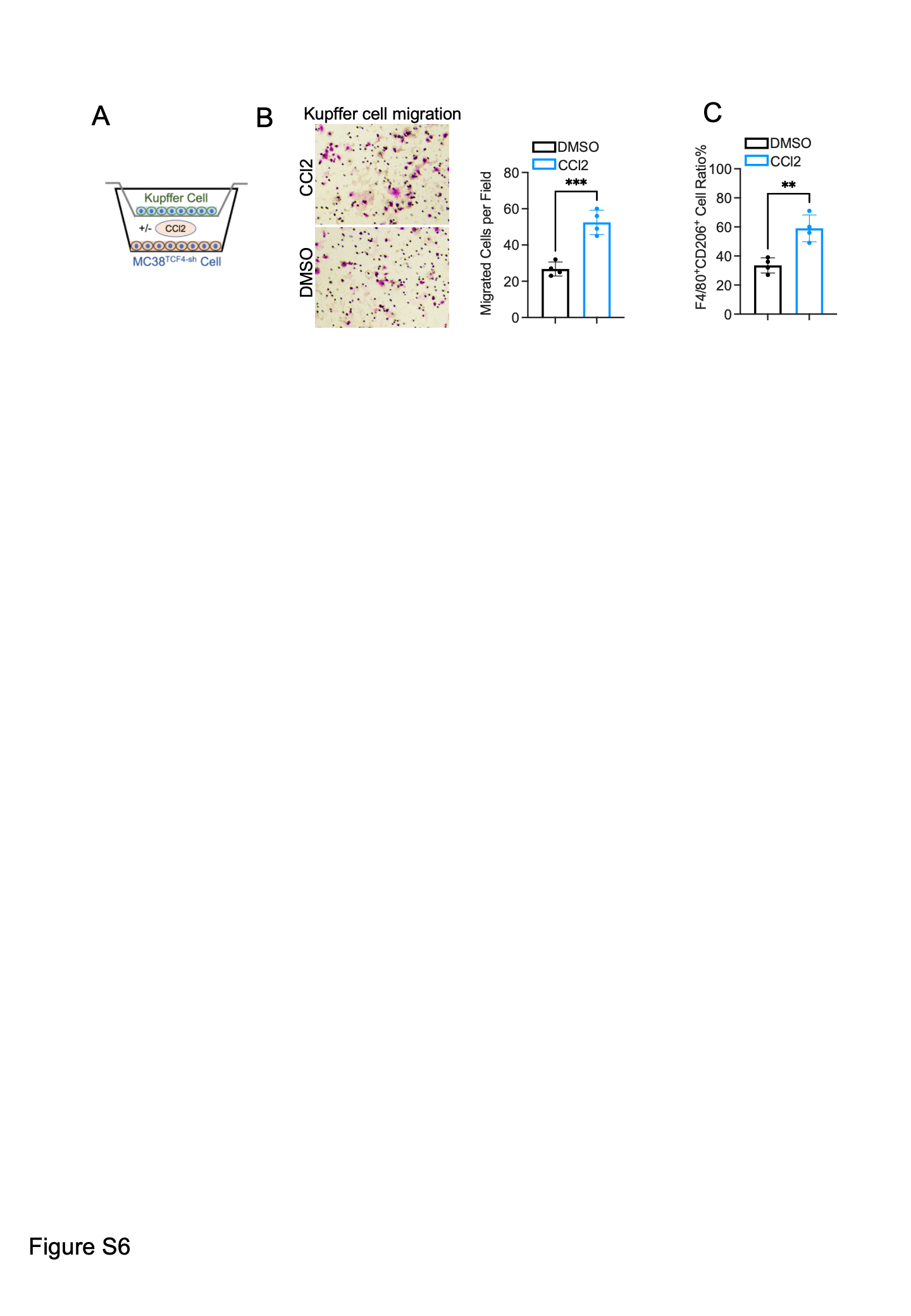

Supplement: Supplementary file 7 — Figure S6 [file 41419_2021_4166_MOESM7_ESM.tif]

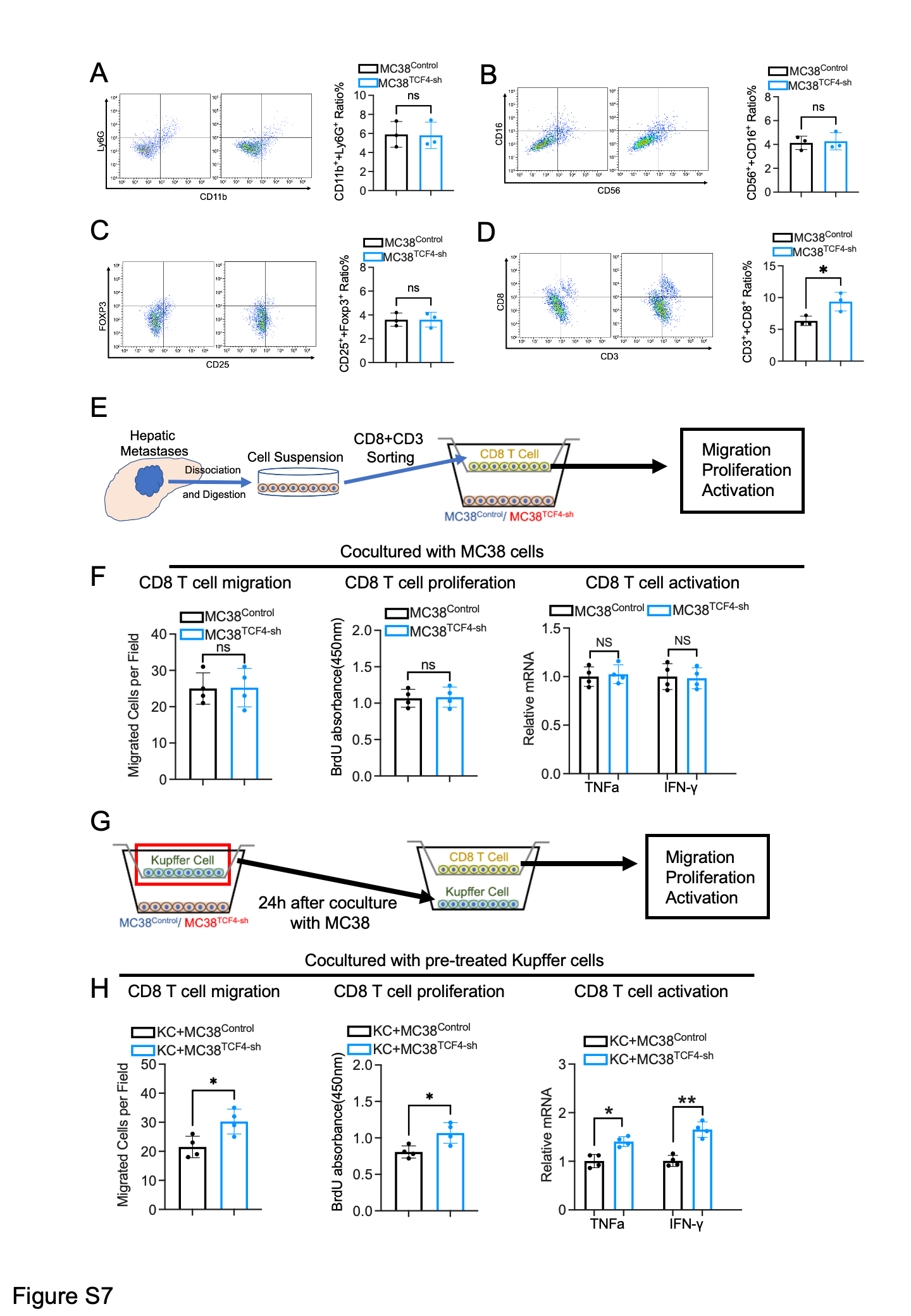

Supplement: Supplementary file 8 — Figure S7 [file 41419_2021_4166_MOESM8_ESM.tif]

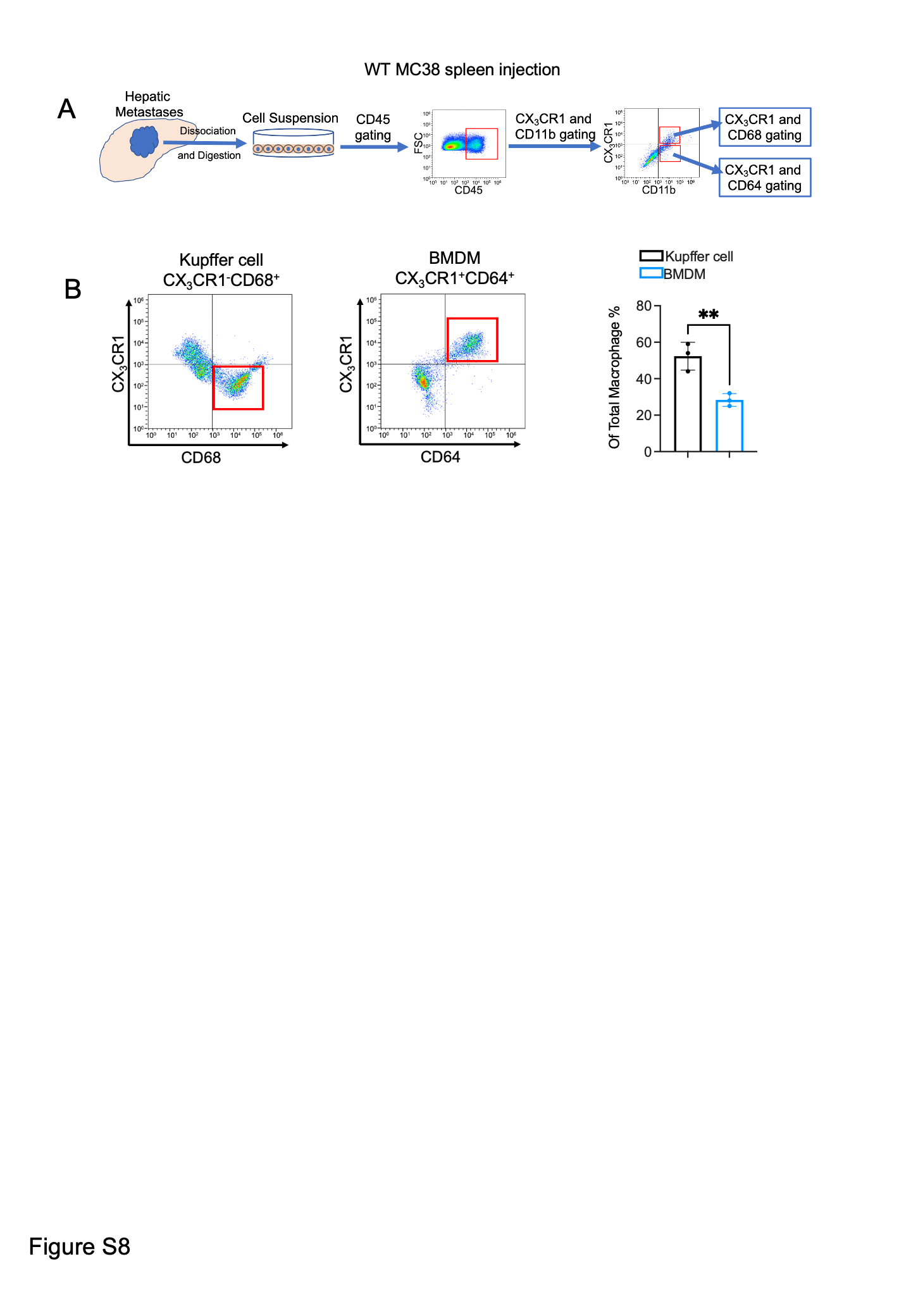

Supplement: Supplementary file 9 — Figure S8 [file 41419_2021_4166_MOESM9_ESM.tif]
